# A Systematic Review: Is *Aedes albopictus* an Efficient Bridge Vector for Zoonotic Arboviruses?

**DOI:** 10.3390/pathogens9040266

**Published:** 2020-04-07

**Authors:** Taissa Pereira-dos-Santos, David Roiz, Ricardo Lourenço-de-Oliveira, Christophe Paupy

**Affiliations:** 1 MIVEGEC, Univ. Montpellier, IRD, CNRS, 34090 Montpellier, France; david.roiz@ird.fr; 2 LATHEMA, Instituto Oswaldo Cruz, FIOCRUZ, Rio de Janeiro-RJ 4364, Brazil; lourenco@ioc.fiocruz.br

**Keywords:** *Aedes albopictus*, emerging diseases, vector competence, spill-over, blood-feeding, bridge vector, arboviruses, mosquito

## Abstract

Mosquito-borne arboviruses are increasing due to human disturbances of natural ecosystems and globalization of trade and travel. These anthropic changes may affect mosquito communities by modulating ecological traits that influence the “spill-over” dynamics of zoonotic pathogens, especially at the interface between natural and human environments. Particularly, the global invasion of *Aedes albopictus* is observed not only across urban and peri-urban settings, but also in newly invaded areas in natural settings. This could foster the interaction of *Ae. albopictus* with wildlife, including local reservoirs of enzootic arboviruses, with implications for the potential zoonotic transfer of pathogens. To evaluate the potential of *Ae. albopictus* as a bridge vector of arboviruses between wildlife and humans, we performed a bibliographic search and analysis focusing on three components: (1) The capacity of *Ae. albopictus* to exploit natural larval breeding sites, (2) the blood-feeding behaviour of *Ae. albopictus*, and (3) *Ae. albopictus’* vector competence for arboviruses. Our analysis confirms the potential of *Ae. albopictus* as a bridge vector based on its colonization of natural breeding sites in newly invaded areas, its opportunistic feeding behaviour together with the preference for human blood, and the competence to transmit 14 arboviruses.

## 1. Introduction

The human alteration of Earth’s natural systems has become a great concern and a threat to human health. Indeed, these changes are likely to drive most of the global disease burden over the coming century [1]. During the last decades, the burden of emerging infectious diseases has increased to represent a substantial threat to global health, security, and economy growth. About 75% of emerging infectious diseases are zoonotic diseases, mostly of wildlife origin [2,3]. The risk of zoonotic emergences is considered high in tropical forest regions associated with a range of facilitating factors, particularly high vertebrate species diversity and agricultural land use changes [4]. Understanding the mechanisms of disease emergence allows the development of early detection and control programs for reducing disease incidence and economic burden [5].

Zoonotic pathogens can be transmitted from animals to humans directly, or indirectly when arthropod vectors are needed to accomplish their life cycle. Zoonotic vector-borne diseases are maintained in enzootic cycles, but can be transmitted from animal reservoir populations to sympatric human populations or to domestic animals during “spill-over events”, and also from humans to animals during “spill-back events” [2,6]. The global emergence of vector-borne diseases is helped by international travel and trade, after their local emergence has been driven by a combination of environmental changes that are not yet completely understood [7]. Therefore, research is needed to determine the potential of these pathogens to emerge in the future, and to identify critical geographic areas where early warning systems must be put in place to mitigate the pathogen’s impact on human health [8].

Here, we focused on zoonotic arboviruses (arthropod-borne viruses) transmitted by mosquitoes that are part of enzootic cycles evolving in wildlife or domestic animals, independently of mankind. Animals might act as amplification hosts for spill-over events to humans [9], mainly in tropical forest environments [10]. Some arboviruses, such as those causing epidemic *Aedes*-borne viral diseases (dengue, chikungunya, and zika), have adapted to epidemic cycles in which viremic humans became the source of infection in urban areas where *Aedes aegypti,* and to a lesser extent, *Aedes albopictus* [11,12] ensure person-to-person transmission [13]. The burden of *Aedes*-borne diseases is dramatic. For instance, dengue incidence has increased by 30 times over the last 50 years, with about 390 million infections reported annually worldwide [14,15]. Dengue and chikungunya outbreak waves have resulted in several million cases in the Southwest Indian Ocean region, India, and the Americas [16]. Zika virus (ZIKAV) disease emerged in 87 countries (or territories) [17]. ZIKAV infection during pregnancy can cause microcephaly in newborns and is becoming a major threat due to its long-term sanitary and economic impacts, especially in Latin America [18]. Although these infection outbreaks are caused by independent urban cycles, enzootic cycles still remain essential sources of pathogens and/or vectors that can be introduced, adapt, and disperse, causing new severe threats [2], as exemplified by the recent re-emergence of Yellow Fever Virus (YFV) in Brazil, Angola, and the Democratic Republic of Congo [19,20]. For YFV, spill-over events from non-human primates that involve mosquito bridge vectors have been described in tropical Africa (e.g., involving *Aedes africanus* or *Aedes furcifer*) [21] and in America (involving mosquito species from the *Haemagogus* and *Sabethes* genera) [20]. After its introduction in the Americas, YFV has efficiently spilled back into sylvatic cycles via bridge vectors. In African villages or cities, YFV transmission is supported by epidemic vectors, such as *Ae. aegypti* [21,22]. These data indicate that mosquito bridge vectors play key roles in the early processes leading to the emergence of enzootic viruses, before the urban transmission cycles [6,8].

We define a bridge vector as an “appropriate hematophagous arthropod” that ensures the biological transmission of a pathogen across different landscapes and its circulation between enzootic, domestic animal, and human hosts. In the absence of a bridge vector, pathogen transmission generally remains restricted to a specific area within the enzootic or epidemic cycle and among hosts/reservoirs. Bridge vectors are the key that interconnects animal reservoirs to new vertebrate hosts, including humans, and that allows both spill-over and spill-back events. For this study, we considered that bridge vectors show several bio-ecological traits that influence the shifting risks of pathogen transfer and that are mainly related to their ecological distribution, blood feeding behaviour, and vector competence. Regarding ecological habitats, high ecological and physiological plasticity favours the vector dispersal and its establishment (breeding in specific microhabitats) across different ecosystems, landscapes, or habitats (e.g., forest/rural/urban, forest/savannah, ground/canopy, natural/anthropic larval breeding and adult resting sites). Regarding blood feeding behaviour, low specificity in blood-meal sources and opportunistic feeding behaviour involving multiple hosts increases the probability of contact between the vector and different animal reservoirs, and thus interspecies pathogen transfer. This probability also depends on the vector and host density and on the host’s defensive behaviour. Regarding vector competence, for the biological transmission of a pathogen after its acquisition on an infected vertebrate, a bridge vector must be able to ensure its replication/multiplication, dissemination, and transmission to subsequently bitten vertebrates. Arthropod species competent for a large panel of pathogens or with high vector competence for one pathogen represent particularly suitable candidates to act as (bridge) vectors.

Here, we evaluated the potential role of the Asian tiger mosquito *Ae. albopictus* as a bridge vector. This invasive vector species originates from Asian tropical forests, but nowadays is present in all continents [23], and has become a major public health issue. In its native area, sylvatic *Ae. albopictus* populations complete their biological cycle by exploiting wild animals as blood sources, and natural water collection points (e.g., tree holes, bamboo stumps, or rock holes) as oviposition sites in the woods [24], particularly at the forest edge [24]. The capacity to colonize artificial man-made containers (together with desiccation-resistant and diapausing eggs) led to its “domestication”. Its ecological plasticity to several habitats, its passive dispersion through the global transport of tires and inside cars [25], and the inefficiency of control programmes have allowed *Ae. albopictus* to become one of most invasive species worldwide [23,24]. In native and newly colonized areas, it has been found in urban, rural, and forest habitats; however, unclear information is available on its natural breeding sites and its presence in forested environments. Moreover, its presence in natural breeding sites in the invaded territories has not been analysed. In general, *Ae. albopictus* is considered an opportunistic feeder that is attracted to mammals, particularly humans, rather than other hosts [26,27]. However, to our knowledge, a detailed and quantified analysis of its host preferences has never been done.

In relation to epidemic virus transmission, *Ae. albopictus* has been considered the vector for the chikungunya virus (CHIKV), dengue virus (DENV), and ZIKAV in Gabon and Central Africa [28,29], for DENV and CHIKV in la Réunion island [30], and for CHIKV in Madagascar and Mayotte [30,31]. In Europe, it has been incriminated in Italy and France during CHIKV and DENV outbreaks [32,33] and in Japan in DENV transmission [34]. Moreover, this mosquito represents a potential risk of outbreaks in many other areas, for example, in Brazil and USA where *Ae. albopictus* is widespread [35,36,37,38]. Different studies have shown that *Ae. albopictus* can develop infection from up to 32 arboviruses [16,23,36]; however, to our knowledge, its ability to transmit any of them has not been clearly demonstrated yet.

In this work, we hypothesized that *Ae. albopictus* may have an active role as a bridge vector for the transfer from vertebrate hosts to humans (spill-over events) and therefore, in the emergence of enzootic arboviruses. To test this hypothesis, we reviewed and quantified: (1) *Ae. albopictus’* capacity to exploit natural water collections as larval breeding sites (as a proxy for its establishment in rural/sylvatic/forested areas) in native or invaded regions; (2) its feeding behaviour with regard to humans, domestic, or wild animals (as a proxy for the contact between vertebrate hosts and humans); and (3) its vector competence, tested experimentally for different arboviruses and natural infections reported from the field in mosquitoes (as a proxy for its potential for virus transmission in the field). Finally, we discuss the potential spill-over transmission risk from vertebrate hosts to humans and the methodological issues and knowledge gaps that need to be tackled.

## 2. Results

### 2.1. Natural Breeding Sites

**Figure 1:**
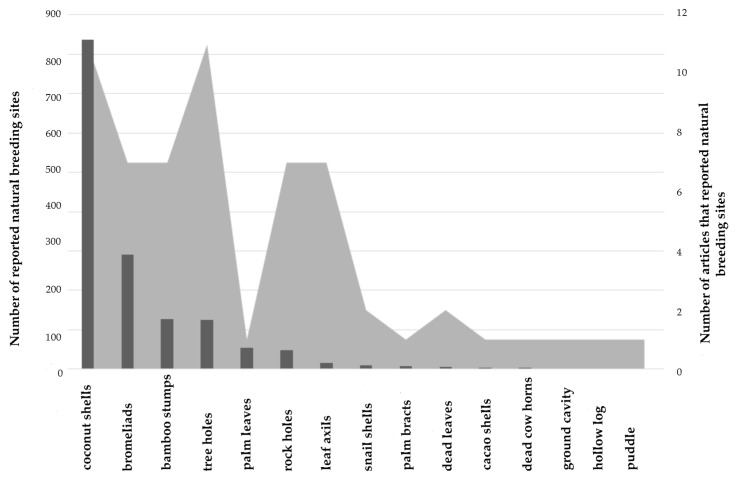
Natural larval breeding sites exploited by *Ae. albopictus*. Number of reported natural breeding sites (black bars) and number of articles that reported natural breeding sites (grey areas).

Based on the literature (see Methods and Supplementary Tables S1 and S2), we found 27 articles that quantified the number and type of natural breeding sites exploited by *Ae. albopictus* in areas where the species is considered native (n = 10 articles) or invasive (i.e., colonized areas) (n = 17 articles). Preimaginal stages of *Ae. albopictus* were mainly detected in coconut shells (54.7%) [37,38,39,40,41,42,43,44,45], bromeliads (19%) [46,47,48,49,50], bamboo stumps (8.3%) [39,40,51,52,53,54], tree holes (8.2%) [37,42,43,51,53,54,55,56,57,58,59], palm leaves (3.6%) [51], rock holes (3.2%) [37,42,43,51,53,57,60], leaf axils (1%) [39,40,42,61], and sporadically (<1%) in other natural breeding sites, such as snail shells [43,53], palm bracts [53], dead leaves [37,43], cacao pods on the ground [43], dead cow horns [43], puddles [62], ground cavities [46], and hollow logs [53] (Figure 1).

Coconut shells and tree holes were more often reported (11 articles each), followed by bamboo stumps, bromeliads, rock holes, and leaf axils (7 articles each), and finally, the other natural breeding sites (1–2 articles each). In native areas, most of the reported natural breeding sites were coconut shells (83%), followed by bamboo stumps (11%), tree holes (5%), leaf axils (1%), and rock holes (1%). In colonized areas, a great diversity of breeding sites was reported: bromeliads (50.8%), tree holes (13%), palm leaves (9.6%), rock holes (8.4%), coconut shells (8%), bamboo stumps (3.8%), leaf axils (1%), palm bracts (1.2 %), snail shell (1.7%), and others (<1% each).

### 2.2. Feeding Behaviour

**Figure 2:**
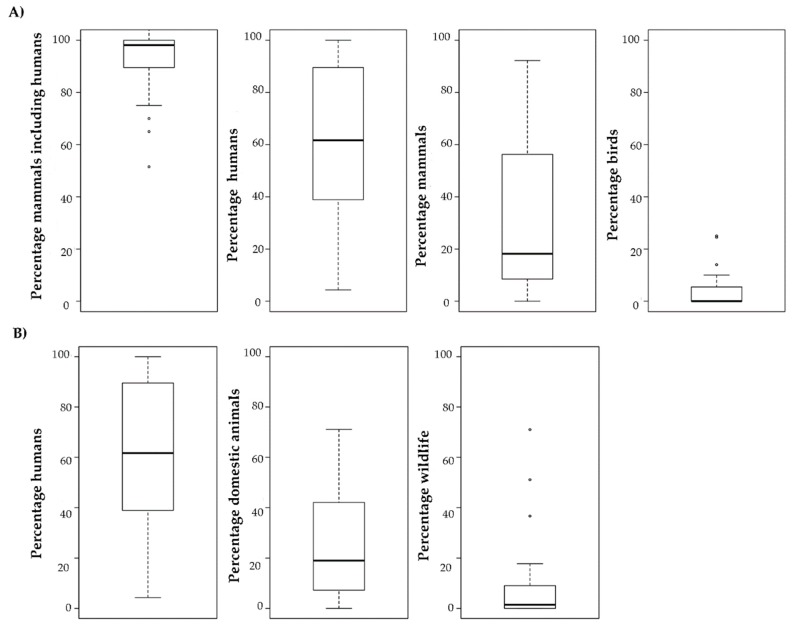
Boxplots showing the host feeding preferences (i.e., percentage of bites) of *Ae. albopictus* without taking into account host availability. (**A**) Mammals, humans, non-human mammals, and birds; (**B**) Humans, domestic animals, and wildlife. Black line: median.

Our quantification of the feeding behaviour indicates that *Ae. albopictus* has been mainly reported as a species that prefers mammals (including humans) as blood sources (mean and standard deviation: 92.0% ± 8), compared with birds (8% ± 8) and other animals (3.7% ± 1.7) [26,27,63,64,65,66,67,68,69,70,71,72,73,74,75,76,77,78,79,80,81,82] (Figure 2A). Among mammals, blood meals were mainly from humans (60%) than non-human species (30%). From an “interspecies risk of transfer” perspective, it is relevant to note that *Ae. albopictus* seems to be biting domestic animals (25%) more frequently than wildlife animals (10%) (Figure 2B). Importantly, there is huge variability in the percentage of human blood meals in the different studies.

Among domestic and peri-domestic animals, dogs, rodents, and rabbits were reported as the main blood sources for *Ae. albopictus*, followed by cats, bovines, chickens, horses, and pigs (Supplementary Figure S1). When classified according to the biological family of blood sources, *Ae. albopictus* can feed on 28 different host biological families, and preferentially on animals belonging to Hominidae (60%), Muridae (15%), Canidae (12%), and Phasianidae (10%) (see Table 1 for detailed information and Supplementary Table S3 for bibliographical information).

### 2.3. Arbovirus Transmission

In the literature search, in addition to the epidemic DENV (serotypes 1, 2, 3, and 4), CHIKV and ZIKV, we found reports on experimental infections of *Ae. albopictus* with the following 36 arboviruses: Arumowot (AMTV) [83], Bujaru (BUJV) [83], Bussuquara (BSQV) [84], Cache Valley (CVV) [85], Chandipura (CHPV) [86], Chilibre (CHIV) [83], Eastern Equine Encephalomyelitis (EEEV) [87,88,89,90], Getah (GETV) [91], Icoaraci (ICOV) [83], Ilheus (ILHV) [92], Itaporanga (ITPV) [83], Jamestown Canyon (JCV) [93], Japanese Encephalitis (JEV) [92,94,95,96], Karimabad (KARV) [83], Keystone (KEYV) [92,93], Kokobera (KOKV) [92], Kunjin (KUNV) [92], La Crosse (LACV) [92,93,97,98,99], Mayaro (MAYV) [100], Oropuche (OROV) [100], Orungo (ORUV) [101], Pacui (PACV) [83], Potosi (POTV) [102,103,104], Rift Valley fever (RVFV) [105,106], Ross River (RRV) [107,108], Salehabad (SALV) [83], San Angelo (SA) [84,92,109], St. Louis encephalitis (SLEV) [110], Tensaw (TENV) [111], Trivittatus (TVTV) [93], Uganda S. (UGSV) [92], Urucuri (URUV) [83], Usutu virus (USUV) [112], Venezuelan equine encephalitis (VEEV) [113,114,115], West Nile virus (WNV) [116,117,118,119,120,121,122,123,124,125], and YFV [108,126,127,128,129,130] (see Supplementary Table S4 for bibliographical information). However, besides the addition to the epidemic DENV (serotypes 1, 2, 3 and 4) [131], CHIKV, and ZIKV [28], natural infections of *Ae. albopictus* were only reported for eight viruses: CCV [85,132], EEEV [133], KEYV [133], LACV [99,132,134,135], POTV [102,132,136], TENV [133], USUV [112], and WNV [118,119,120] (see Supplementary Table S5 for bibliographical information). These infections were detected by virus isolation on cell lines, immunological or molecular methods (Vero cells, direct or indirect immunofluorescence, polymerase chain reaction). These infections provide evidence of contact between *Ae. albopictus* and the hosts of these viruses, but do not necessarily indicate their biological transmission by this mosquito. On the other hand, for the BSQV, ILHV, KOKV, KUNV, and UGSV arboviruses, only intrathoracic injection experiments were carried out to investigate transovarian transmission between different generations. Supplementary Table S6 gives information on the taxonomic classification of these viruses, their geographic distribution, their natural host family (i.e., vertebrate host family in which the virus was isolated or in which serological evidence was found), the mosquito species from which the virus was isolated, and the detection method in *Ae. albopictus*.

Among studies on *Ae. albopictus* vector competence, we found important variations concerning the methodology used to perform the infection (intra-thoracic inoculation of viruses, oral challenge using infected blood meals or infected animals), the mosquito strains, the viral strains and the virus loads used, the conditions of mosquito incubation (e.g., time, temperature), and the methods used to determine mosquito infection and transmission efficiency. Concerning the virus inoculation methodology, intra-thoracic injection was used for 11 viruses to assess vector infection, and oral infection was performed using infected hosts (n = 11 arboviruses), or membrane feeding methods (n = 11 arboviruses).

The mean infection values in *Ae. albopictus* after infection by intrathoracic injection greatly varied in function of the tested virus, and ranged from 100% ± 0 (AMTV, BUJV, and PACV) to 37.5% ± 17.67 (ORUV). Among these viruses, the transmission rate after intrathoracic injection was estimated only for ORUV (37.5% ± 17.67) and RVFV (15.9% ± 7.3). The mean infection rate (IR) in *Ae. albopictus* that fed directly on infected vertebrate hosts or on an infectious artificial blood-meal through a membrane also hugely varied, from 100% ± 0 for GETV to 6.6% ± 5.2 for OROV. The mean Dissemination Efficiency (DE) in *Ae. albopictus* varied from 89.85% ± 5.9 for POTV to 4.06% ± 1.32 for MAYV. The mean Transmissions Rates (TR) in *Ae. albopictus* varied from 82.7% ± 11.5 (WNV) to 7.7% ± 0 (JCV). Finally, the mean Transmission Efficiency (TE) by *Ae. albopictus* varied from 68.6% ± 18.6 (WNV) to 3.5% ± 0.69 (MAYV).

**Figure 3:**
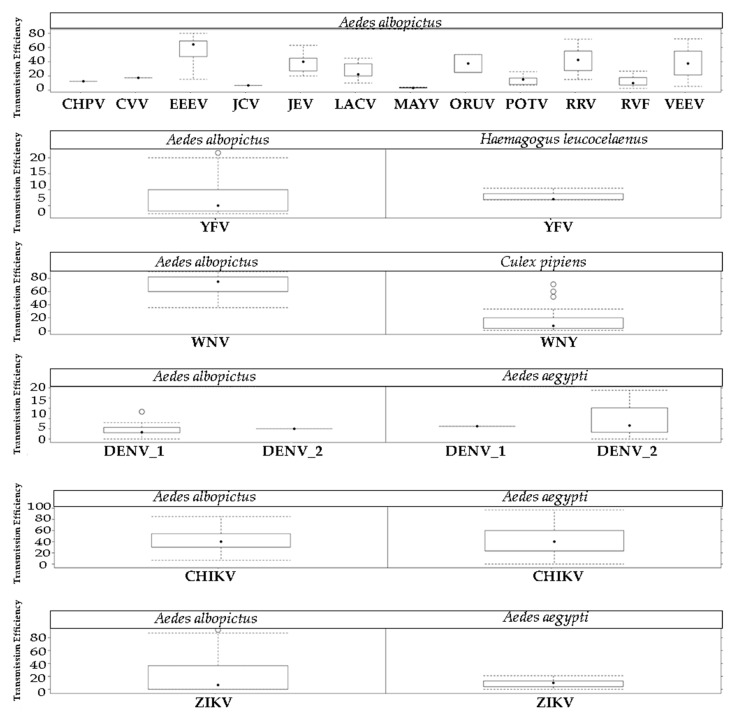
Transmission efficiency across studies that evaluated *Ae. albopictus* vector competence for different arboviruses. Bridge Vector*Virus and Epidemic Vector*Virus pairs were added to compare the transmission efficiency.

Whatever the methodology used for the experimental infection, transmission was confirmed for 14 viruses. Six displayed a mean TE higher than 30% (WNV, EEEV, RRV, JEV, VEEV, and ORUV), and five had a mean TE between 10% and 30% (LACV, CVV, POTV, CHPV, and RVFV). The mean TE for YFV, JCV, and MAYV was below 10%. All TE rates in *Ae. albopictus* (using both experimental infections with infectious animals and infectious artificial blood meals) are summarized in Figure 3, without taking into account the different mosquito populations used, the viral loads, or genotypes. For more details on the infection parameters (IR, DE, TR, and TE) obtained using the different inoculation methods, see Table 2.

Comparison of IR, DE, and TE (see Methods and Supplementary Table S7) values calculated for known efficient bridge vectors infected with different arboviruses, and those for *Ae. albopictus* (Table 3) showed that the YFV TE rate for *Ae. albopictus* (7.68% ± 5.9) was similar to the rate calculated for *Haemagogus leucocelenus* [127] (8.08% ± 2.0). Conversely, the TE rates varied more for WNV: 68.6% ± 18.6 for *Ae. albopictus* and 13.49 ± 14.8 for *Culex pipiens* (a primary vector of WNV in the field) [117,124,137,138,139,140,141,142,143,144,145,146]. Moreover, *Ae. albopictus* and *Ae. aegypti* (a recognized epidemic vector) showed similar TE rates for CHIKV [126,147,148,149,150,151,152], DENV-1 [153,154,155,156,157], and DENV-2 [126,147,154,155,157,158,159], but *Ae. aegypti* was more efficient at transmitting ZIKV [126,160,161,162,163,164,165,166,167,168,169,170,171] and YFV [127,128,172,173,174,175].

## 3. Discussion

In the present work, we tried to understand the potential role of the Asian tiger mosquito *Ae. albopictus* as a bridge vector that might favour the transfer of zoonotic arboviruses from enzootic or domestic hosts to humans and vice-versa. To this aim, we evaluated its ability to colonize natural breeding sites in newly invaded and native areas, its appetence for animal blood sources, and its global efficiency for transmitting arboviruses. This mosquito species was described as capable of developing infection from a large number of arboviruses in laboratory conditions [36]. However, based on the published evidences of vector competence, we found that transmission by *Ae. albopictus* is proven only for 14 of them, without considering the epidemic *Aedes*-borne CHIKV, DENV (4 serotypes), and ZIKAV.

In relation to the capacity of *Ae. albopictus* to establish in natural areas (rural/sylvan environments), tree holes were described as the most common natural breeding sites, although it has been detected also in bamboo stumps, and more sporadically in rock holes and plant axils [24]. Our analysis indicates that coconut shells, bromeliads, and bamboo stumps might be as common as tree holes, whereas rock holes and leaf axils of other plants are less frequently used. These results might be biased due to differences across studies related to sampling efforts and the environmental characteristics of sampled areas. Therefore, they should be confirmed by comparisons with larval sampling in natural and artificial breeding sites in natural areas and forest edges. Moreover, when possible, the productivity in these habitats should be described and compared by pupal sampling, with the same methodology used for quantifying the productivity of anthropic containers in urban areas [176]. For example, a study in Rio de Janeiro showed that the percentage of *Ae. albopictus* larvae in bromeliads corresponded to 0.18% of all sampled larva, demonstrating the low productivity of this breeding place [48]. However, studies describing the productivity of natural breeding sites in the natural environment or at an interface between natural and man-modified environments are lacking. In native forested areas, natural containers of larvae (tree holes, bamboo stumps, rock holes) were observed at the forest edge, like in a colonized forested area. Breeding sites in the deep forest have never been detected for this species [24,27].

Our results also confirmed the opportunistic feeding behaviour of *Ae. albopictus* and its strong preference for mammals, especially humans (humans = 60%, non-humans = 30%) compared with other groups, such as birds (4%). *Ae. albopictus* can feed on 28 different biological families. Reports on *Ae. albopictus* biting on any primates other than man were lacking until very recently. Specifically, a study described *Ae. albopictus* probing on a howler monkey that had just died due to YFV and was lying on the forest edge in Brazil [177]. This mosquito also bites domesticated animals—Muridae, Canidae, Phasianidae, Herpestidae, and Bovinae. Several studies suggested this opportunism. For instance, laboratory experiments on the host choice showed that this mosquito preferentially bites humans compared with other animals [30]. This opportunism was confirmed in studies on blood-fed mosquitoes collected in the field [27,30,69,78]. From our literature analysis, birds appeared as a non-preferential host group. Based on the reported proportion of blood meals, domestic and peri-domestic animals (25%) should be considered more relevant than wildlife (10%) as sources of zoonotic pathogens for *Ae. albopictus*. However, a limited number of studies were carried out in natural habitats where wildlife is abundant. Therefore, additional research is needed in natural areas to precisely describe the blood feeding patterns of *Ae. albopictus* and its interaction with wildlife. If possible, the availability of vertebrate hosts should be taken into account by using field census procedure and by calculating indexes of feeding preferences [178]. Such approaches should prevent the underestimation of the *Ae. albopictus’* potential to transmit pathogens from domestic/sylvatic vertebrate hosts to humans, but also from domestic to sylvatic vertebrate hosts, and vice versa. Our analysis also highlighted a huge variability in the proportion of human blood meals. This is a relevant factor for calculating the vector capacity, the disease reproduction rate (Ro), and the spill-over risk that may be determined by several parameters [178].

Concerning vector competence, this species was suggested as a potential vector for many viruses. It is important to emphasize that the mean TE values of enzootic viruses, such as WNV (68.6% ± 18.6), EEEV (57.16% ± 20.14), RRV (41.39% ± 16.5), JEV (39.3% ± 13.5), VEEV (38.1% ±), LACV (27.3% ± 12.87), CVV (17.4% ± 0), and POTV(14.6% ± 7), were higher or comparable with those reported for epidemic viruses, such as DENV-1 (6.25 ± 0), DENV-2 (10.13 ± 12.28), YFV (7.68 ± 5.9), ZIKV (9.21 ± 6.9), CHPV (12.5% ± 0), YFV (8.2% ± 6), JCV (6.6% ± 0), RVFV (5.2% ± 3.9), and MAYV (3.5% ± 0.69). The large difference in TE rates between enzootic and epidemic viruses is a reflection of the techniques employed to assess parameters. Most of the analysis on enzootic viruses were performed mainly in the 1990s and up to the beginning of the 2000s. Conversely, epidemic viruses were analysed using more precise techniques during the last 5 years. Despite the biases of the older methodologies, *Ae. albopictus* presented a high TE rate for enzootic arboviruses; therefore, it might transmit these viruses if taken from viremic natural vertebrates.

Comparing the vector competence of *Ae. aegypti* and *Ae. albopictus* for different epidemic viruses did not allow for a conclusion that there is a difference in their TE rates for ZIKV, CHIKV, DENV-1, and DENV-2. However, for bridge vectors*virus pairs, WNV TE was higher for *Ae. albopictus* than for *Cx. pipiens,* contrary to what was expected. Although the WNV transmission efficiency rate by *Ae. albopictus* is high in experimental conditions, this species has never been incriminated as a WNV vector in the field, possibly due to its low propensity to bite birds. *Ae. albopictus* presented similar TE rates as *Hg. leucocelenus,* a primary YFV vector within and at the edges of Brazilian forests [27,179]. However, few studies have been carried out to assess *Hg. leucocelenus* vector competence. In general, the contribution of laboratory studies for assessing the role of vector(s) in natural environments is limited.

Based on vector competence and blood meal studies, we conclude that *Ae. albopictus* could act as a bridge vector for many viruses (e.g., WNV, EEEV, ORUV, RRV, YFV, JEV, VEEV, LACV, RVFV, CVV, CHPV, JCV, and MAYV) with a potential risk for disease emergence. One of our goals was to identify in a quantitative way the viruses with a higher risk of emergence, and to develop an analysis to quantify the relative risk of transfer to humans of each enzootic arbovirus that can be efficiently transmitted by *Ae. albopictus* in laboratory conditions. The methodology used was based on two previous published works [180,181] that quantified the risk of WNV transfer by *Culex* mosquitoes. We then calculated the relative risk of *Ae. albopictus*-mediated virus transfer from its natural hosts to humans using a simplified version of Kilpatrick’s equation (see Supplementary Information for more details concerning the methodology used and Figure S2) that takes into account *Ae. albopictus* vector competence for a given virus (i.e., TE), and the mean relative feeding frequencies on humans (FHi) and on animal hosts (FAi). Unfortunately, this analysis was hindered by the limited information available on the enzootic/sylvatic reservoirs of several of these arboviruses (some hosts remain unknown or are not sufficiently identified). Moreover, some viruses have many potential reservoirs, and their objective weighting is difficult. Additionally, data on *Ae. albopictus* propensity to bite a given animal reservoir species are often lacking (e.g., primates). Consequently, only biting frequencies at animal family levels could be used, leading to overly unreliable and speculative risk transfer estimates. Therefore, we chose not to include them here, although these estimates are crucial to better assess the risk of spill-over and emergence of enzootic arboviruses in relation with the secondary invasion of *Ae. albopictus* in forested areas.

Another important limitation of the present work is the great methodological variation and the lack of standardization of the protocols used to assess the vector competence of *Ae. albopictus*. Vector competence for arboviruses is influenced by genetic factors in the mosquito population and in the virus strain, such as the geographical genetic origin of the vector population or the interaction between the vector and arbovirus genotype [182,183]. Therefore, the intraspecific genetic variability in mosquito species/populations, as well as the intra- and inter-specific variability of arboviruses can affect vector competence and risk estimations. External factors, such as the incubation temperature, can also affect vector competence, and consequently the transmission and analysis of the risk [184].

Other factors interfering with the vector competence results are the way of ingesting the virus-infected blood (in vivo or in vitro), the viral load concentration, and the sensibility of the method used to detect the virus in the mosquito body or saliva. We are aware that our study is limited due to the methodological differences of the analysed articles, and also because the risk of arbovirus emergence is a multifactorial process and it is actually impossible to estimate the interactions of all factors with the limited evidences available. Thus, more standardized studies of vector competence and blood feeding preferences are necessary. In this sense, the project Infravec2 (https://infravec2.eu) is an important international initiative, and one of its themes is the standardization of methods.

In conclusion, data from the literature show that *Ae. albopictus* can colonize forest environments, and has possible interactions with domestic animals and wildlife, suggesting a risk for interaction with animal viruses. Such a risk is particularly high in areas that are considered to be biodiversity hotspots, such as the Congo and Amazon Basin forests. The presence of *Ae. albopictus* in small towns and hamlets in the Amazon Forest highlights the risk of spill-over of some arboviruses that cause human diseases, such as OROV, YFV, and MAYV [27]. In Brazil, *Ae. albopictus* populations are experimentally competent for YFV transmission, but this has not been confirmed by infecting *Ae. albopictus* [127,185]. In Africa, many arboviruses could be investigated to elucidate their potential transmission and emergence facilitated by *Ae. albopictus*, as done for CHIKV [152]. In the United States, where this mosquito species is widespread, its potential role in LACV, EEEV, WNV, and POTV transmission must be investigated [36,133,135]. In Asia and Oceania, the potential for inter-species transmission of JEV and RRV must be evaluated. It is important to take into account that the risk of arbovirus emergence is dynamic and in continuous evolution because mosquito populations, virus genetics, and the possibility of their contact varies according to time and place, and adaptations could be expected, particularly for invasive pathogens and vectors [186]. For instance, in the Indian Ocean region, the interaction between *Ae. albopictus* and CHIKV led to the selection of a virus strain that infects vectors and can spread around the world more easily. Studies on mutation selection for more susceptible arbovirus strains are still limited, but can be useful for predicting spill-over events [187]. Also, vector competence must be evaluated with as many strains as possible to maximize viral diversity, if possible using strains recently isolated from animals.

Our literature review showed that *Ae. albopictus* is competent for many different arboviruses, is present in natural habitats and forest edges, and can feed on several animal groups [30]. All these features make of *Ae. albopictus* a potential bridge vector of several emerging arboviruses (at least 14 viruses [23,36]), thus increasing the risk of spill-over and spill-back events. We hope that our approach will encourage more research to disentangle this risk in the field and the laboratory, with the aim of preventing the emergence of zoonotic diseases and reducing potential health and economic burdens, particularly for vulnerable populations.

## 4. Material and Methods

### 4.1. Natural Breeding Sites

First, a literature search was done in Google Scholar to identify articles reporting the presence of *Ae. albopictus* in natural larval breeding sites and their types, using the keywords “Natural Breeding sites *Aedes albopictus*” or “Oviposition sites *Aedes albopictus*” or “Larval habitats *Aedes albopictus*”. This allowed for the identification of 16 articles [43,44,46,52,54,55,61,62,188,189,190,191,192,193,194] (Supplementary Table S1). From these articles, the main natural breeding sites were listed: bamboo stumps, bromeliads, coconut shells, leaf axils, rock holes, tree holes, snail shells, cacao shells, puddles, dead cow horns, dead leaves, ground cavity, hollow log, palm bracts, and palm leaves. Then, a search on each type of natural breeding site was carried out using PubMed, using the following words: (*Aedes albopictus* [Title/Abstract] AND “Breeding type” [Title/Abstract]). The aim of this search was to quantify the number of articles and the number of detections that described the presence of this mosquito in each of the identified natural breeding sites (Supplementary Table S2). Articles that did not quantify the number of times the breeding sites were found positive were excluded. The bibliographic search was done between August and December 2018.

### 4.2. Feeding Behaviour

A literature search was done in Google Scholar with the key words “blood meal” and “host feeding”, followed by “*Aedes albopictus*” until December 2018. Three studies were excluded because they were considered unreliable: (i) the study by Gingrich and Williams, 2005 [67], which did not test for human blood meals, thus bringing a potential bias into the results; (ii) the study performed in a zoo by Tuten et al., 2012 [195]; and (iii) the study by Hess et al., 1968 [196] that was exclusively carried out in a bird area on Hawaii Island. Finally, 22 studies were selected (see references and details for each of them in Supplementary Table S3) to build a database of blood feeding preferences, based on the *Ae. albopictus* biting frequency for each host species, biological family, or group of vertebrate hosts (human, mammals, birds, domestic animals, wild animals). The database was used to quantify the relative importance as a blood meal of each host group and of specific hosts, based on the reported blood meal sources identified using different techniques (DNA sequencing, ELISA blond meal analyses, agarose gel precipitin). Then, these preferences were analysed independently of the host availability, which was quantified in very few studies.

### 4.3. Arbovirus Transmission

First, all referenced arboviruses that might be transmitted by *Ae. albopictus* were selected using the arbocat database from Centers for Disease Control and Prevention (CDC) (https://wwwn.cdc.gov/arbocat/VirusBrowser.aspx). Then, Google Scholar and PubMed were searched with the key words “Virus name” and “Vector Competence”, followed by “*Aedes albopictus*”. Among the 49 articles obtained with this search, articles containing data on virus detection/isolation from field-collected mosquitoes, and data on vector competence parameters, including “susceptibility”, “infection, dissemination”, or “transmission rates” were selected (see Supplementary Table S4 showing the viruses and the bibliographic references). Data from each article were used to calculate the infection rates as the number of mosquitoes showing virus infection in the gut divided by the number of mosquitoes fed with infected blood x 100. Dissemination efficiency was calculated as the number of mosquitoes with viruses disseminated in the legs, wings, or head divided by the number of mosquitoes fed with infected blood x 100. Transmission rates were calculated as the number of mosquitoes that could deliver the virus with saliva (detection of virus in mosquito saliva, or demonstration of transmission using animal hosts exposed to infected mosquito bites) divided by the number of mosquitoes with viruses disseminated in the legs, wings, or head (body) × 100. Transmission efficiency was calculated as the number of mosquitoes that could deliver the virus with saliva (detection of living viruses or viral genome in mosquito saliva, or demonstration of transmission using animal hosts exposed to infected mosquito bites) divided by the number of mosquitoes fed with infected blood [168]. In the present work, infection performed from intrathoracic assays corresponds to mosquitoes that after intrathoracic injection, were detected with the virus after a 7–10 day incubation period. For this detection, the ground mosquito suspension was inoculated in rats, or the presence of the virus quantified by assays in Vero cell cultures. After intrathoracic injection, infected mosquitoes may transmit the virus to another animal. Some articles only described transovarial transmission tested after intrathoracic infection. These works demonstrated *Ae. albopictus* susceptibility to develop infection by a given arbovirus. However, these articles did not quantify the infection and transmission rates.

To compare the results, the same bibliographic search was performed to find the vector competence values reported for efficient bridge vector–virus pairs, such as *Culex pipiens* * WNV and *Haemagogus leucocelenus* * YFV, and for epidemic vector–virus pairs, such as Aedes aegypti * YFV, *Aedes albopictus* *DENV_1, *Aedes albopictus* *DENV_2, *Aedes aegypti* *DENV_1, *Aedes aegypti* *DENV_2, *Aedes albopictus* *CHIKV, *Aedes aegypti* * CHIKV, *Aedes albopictus* *ZIKV virus, and *Aedes aegypti* * ZIKV (Supplementary Table S7). The bibliographic search was done between August 2018 and November 2019.

## Figures and Tables

**Table 1:** Mean biting frequency by *Aedes albopictus* in animals classified according to biological class and family.

Biological Class	Biological Family	Mean Frequency (%)
Aves	Phasianidae	10.08
	Passeridae	7.78
	Anatidae	7.5
	Columbidae	5.83
	Sulidae	2.33
	Thamnophilidae	1.49
	Pycnonotidae	1.39
	Corvidae	1.11
	Ciconiidae	1.0
Mammalia	Hominidae (Humans)	59.83
	Muridae	15.34
	Canidae	11.6
	Herpestidae	9.53
	Bovidae	8.9
	Felidae	8.49
	Leporidae	8.27
	Sciuridae	5.07
	Suidae	4.99
	Didelphidae	4.6
	Equidae	4.39
	Cervidae	4.15
	Muridae/Soricidae	3.43
	Phyllostomidae	2.99
	Procyonidae	2.71
	Furipteridae	1.49
	Cricetidae	0.61
Actinopterygii	Cobitidae	1.11
Amphibia	Salamandridae	2.22

The mean frequencies were calculated using the data found in articles that described different *Ae. albopictus* populations biting different animals in different locations. As these articles do not describe the same biological families, the total mean bite frequency does not correspond to 100%.

**Table 2:** Infection rate, dissemination rate, dissemination efficiency, transmission rate, and transmission efficiency (mean and standard deviation) of *Aedes albopictus* for the indicated arboviruses, according to the inoculation method.

Infection Method	Virus	Infection or Infection Rate		Dissemination Rate		Dissemination Efficiency		Transmission Rate		Transmission Efficiency	
		Mean	SD	Mean	SD	Mean	SD	Mean	SD	Mean	SD
Host feeding	CHPV	25.00	0.00	ND	ND	ND	ND	ND	ND	12.50	0.00
	EEEV	75.36	35.35	85.19	25.66	76.99	33.58	40.00	0	57.17	20.15
	JEV	ND	ND	ND	ND	ND	ND	ND	ND	37.00	9.17
	LACV	ND	ND	ND	ND	ND	ND	ND	ND	23.86	6.69
	MAYV	11.88	3.31	20.00	0.00	4.07	1.32	ND	ND	3.46	0.69
	OROV	6.67	5.20	ND	ND	ND	ND	ND	ND	ND	ND
	POTV	26.26	17.06	ND	ND	ND	ND	ND	ND	ND	ND
	RRV	80.66	23.02	ND	ND	ND	ND	ND	ND	41.40	16.57
	RVFV	69.26	27.24	60.04	6.34	40.72	11.96	15.00	7.07	6.54	4.67
	VEEV	71.20	20.49	89.48	10.48	64.78	22.53	59.94	26.57	38.09	23.53
	WNV	73.41	23.81	94.39	3.91	69.80	23.98	82.72	11.49	68.63	18.62
Intrathoracic injection	AMTV	100.00	ND	ND	ND	ND	ND	ND	ND	ND	ND
	BUJV	100.00	ND	ND	ND	ND	ND	ND	ND	ND	ND
	CHIV	96.88	ND	ND	ND	ND	ND	ND	ND	ND	ND
	ICOV	40.91	ND	ND	ND	ND	ND	ND	ND	ND	ND
	ITPV	81.25	ND	ND	ND	ND	ND	ND	ND	ND	ND
	KARV	94.12	ND	ND	ND	ND	ND	ND	ND	ND	ND
	ORUV	37.50	17.68	ND	ND	ND	ND	ND	ND	37.50	17.68
	PACV	100.00	0.00	ND	ND	ND	ND	ND	ND	ND	ND
	RVFV	ND	ND	ND	ND	ND	ND	ND	ND	15.93	7.35
	SALV	92.86	0.00	ND	ND	ND	ND	ND	ND	ND	ND
	URUV	94.12	0.00	ND	ND	ND	ND	ND	ND	ND	ND
Membrane feeding	CHIKV	58.92	28.23	77.58	22.60	79.06	23.45	53.49	33.98	42.68	23.78
	CVV	56.50	0.00	100.00	0.00	ND	ND	29.60	0.00	17.39	0.00
	DENV-1	60.18	16.01	63.79	23.97	39.56	23.90	8.33	0.00	6.25	0.00
	DENV-2	58.10	30.93	53.12	22.93	34.83	18.81	12.47	13.20	10.13	12.29
	GETV	100.00	0.00	ND	ND	ND	ND	ND	ND	ND	ND
	JCV	96.67	0.00	89.66	0.00	86.67	0.00	7.69	0.00	6.67	0.00
	JEV	91.98	10.72	90.79	14.56	84.63	19.92	ND	ND	40.50	15.98
	KEYV	91.89	0.00	91.18	0.00	83.78	0.00	ND	ND	ND	ND
	LACV	89.72	7.38	86.83	13.70	71.03	22.93	35.84	14.25	29.93	16.75
	POTV	93.55	6.59	96.13	3.21	89.86	5.96	ND	ND	14.67	7.00
	RVFV	10.53	0.00	25.00	0.00	2.63	0.00	100.00	0.00	2.63	0.00
	TVTV	28.00	0.00	85.71	0.00	24.00	0.00	ND	ND	ND	ND
	USUV	64.40	31.2	0.00	0.00	0.00	0.00	0.00	0.00	0.00	0.00
	WNV	32.61	24.53	64.59	25.58	20.33	16.96	ND	ND	ND	ND
	YFV	33.18	21.18	55.52	20.97	20.86	10.90	36.52	26.17	7.68	5.94
	ZIKV	67.19	23.70	38.71	21.76	29.25	22.80	24.62	22.46	9.21	6.91

Infection rate: number of mosquitoes showing virus infection in the gut divided by the number of mosquitoes fed with infected blood x 100. Infection: percentage of mosquitoes in which the virus was detected after 7–10 day of incubation following intrathoracic injection of the indicated virus. For this test, the ground mosquito suspension was inoculated in rats, or the virus presence was quantified by assays in Vero cells. ND: Not described SD: standard deviation AMTV, Arumowot virus; BUJV, Bujaru virus; CHIKV, Chikungunya virus; CVV, Cache Valley virus; CHPV, Chandipura virus; CHIV, Chilibre virus; DENV-1, Dengue virus serotype 1; DENV-2, Dengue virus serotype 2; EEEV, Eastern Equine Encephalomyelitis virus; GETV, Getah virus; ICOV, Icoaraci virus; ITPV, Itaporanga virus; JCV, Jamestown Canyon virus; JEV, Japanese Encephalitis virus; KARV, Karimabad virus; KEYV, Keystone virus; LACV, La Crosse virus; MAYV, Mayaro virus; OROV, Oropuche virus; ORUV, Orungo virus; PACV, Pacui virus; POTV, Potosi virus; RVFV, Rift Valley fever virus; RRV, Ross River virus; SALV, Salehabad virus; SAV, San Angelo virus; SLEV, St. Louis encephalitis virus; TENV, Tensaw virus; TVTV, Trivittatus virus; URUV, Urucuri virus; USUV, Usutu virus; VEEV, Venezuelan equine encephalitis virus; WNV, West Nile virus; YFV, Yellow fever virus; and ZIKV, Zika virus. For BSQV, Bussuquara virus, ILHV, Ilheus virus, KOKV, Kokobera virus, KUNV, Kunjin virus, UGSV, and Uganda S. virus, only transovarial transmission tests were described.

**Table 3:** Comparison of the infection rate, dissemination efficiency, and transmission efficiency (mean and standard deviation) for *Aedes albopictus* and other mosquito vectors.

Mosquito Species	Virus	IR (%)	DE (%)	TE (%)
*Aedes aegypti*	CHIKV	NA	98.3 ± 3.8	42.92 ± 20.19
	DENV-1	37.7 ± 27	34.4 ± 24.9	4.9 ± 4.6
	DENV-2	44.4 ± 33.4	33.3 ± 24.2	5 ± 0
	ZIKV	69.0 ± 27.4	44.0 ± 28.3	20.48 ± 26.87
	YFV	46.4.0±23.6	21.3 ± 19.0	16.5 ± 17.7
*Aedes albopictus*	CHIKV	58.9 ± 28.2	79.0 ± 23.4	42.68 ± 23.7
	DENV-1	60.2 ± 16	39.5 ± 24.2	6.25 ± 0
	DENV-2	58.0 ± 30.9	34.8 ± 18.8	10.13 ± 12.28
	WNV	63.8 ± 29.2	58.1 ± 30.8	68.6 ± 18.6
	YFV	33.1 ± 21.1	20.8 ± 10.8	7.68 ± 5.9
	ZIKV	67.1 ± 23.7	29.2 ± 22.8	9.21 ± 6.9
*Culex pipiens*	WNV	47.7 ± 33.7	30.4 ± 29.7	13.49 ± 14.8
*Haemagogus leucocelenus*	YFV	50.9 ± 4.0	30.06 ± 1.6	8.08 ± 2.0

IR, infection rate; DE, dissemination efficiency; TE, transmission efficiency; CHIKV, Chikungunya virus; DENV-1, Dengue serotype 1; DENV-2, Dengue serotype 2; WNV, West Nile virus; YFV, Yellow fever virus; ZIKV, Zika vírus.

## Supplementary Materials

The following are available online at https://www.mdpi.com/2076-0817/9/4/266/s1, Table S1. List of the 16 articles found by searching Google Scholar to characterize the types of natural breeding sites exploited by *Ae. albopictus,* Table S2. Typology and number of reported natural containers exploited by *Ae. albopictus* from articles found in PubMed, Table S3: List of references used to analyse the host feeding preferences of *Aedes albopictus*, Table S4: List of references that reported infection, infections rate, dissemination rate, dissemination efficiency, transmissions rate or transmission efficiency in *Ae. albopictus* for the indicated arboviruses, Table S5: List of references used to analyse the vector competence of several mosquito-virus pairs: *Aedes aegypti**CHIKV, *Aedes aegypti**DENV-1, *Aedes aegypti**DENV-2, *Aedes aegypti**ZIKV, *Aedes albopictus**CHIKV, *Aedes albopictus**DENV-1, *Aedes albopictus**DENV_2, *Aedes albopictus**ZIKV, *Culex pipiens**WNV, and *Haemagogus leucocelenus**YFV, Table S6: Natural detection or isolation of arboviruses in *Ae. albopictus* from field-collected mosquitoes. CCV, Cache Valley virus; EEEV, Eastern Equine Encephalomyelitis virus; KEYV, Keystone virus; LACV, La Crosse virus; POTV, Potosi virus; TENV, Tensaw virus; WNV, West Nile virus, Table S7: Geographic distribution, vertebrate hosts and potential vectors of arboviruses isolated or tested for vector competence in *Ae. albopictus*. Figure S1. Analysis of the host feeding patterns of *Ae. albopictus* for the different species of domestic animals without taking into account the host availability.
