# Supplementary material for: A Systematic Review: Is Aedes albopictus an Efficient Bridge Vector for Zoonotic Arboviruses?
Source: Pathogens. 2020 Apr 7;9(4):266. doi: 10.3390/pathogens9040266 (PMC7238240; doi:10.3390/pathogens9040266)
Supplement: Supplementary file 1 [file pathogens-09-00266-s001.pdf]

pathogens

MDPI

Article

Supplementary information

A systematic review: Is *Aedes albopictus* an efficient bridge vector for zoonotic arboviruses?

Taissa Pereira dos Santos[1*], David Roiz[1], Ricardo Lourenço-de-Oliveira[2] and Christophe Paupy[1*]

[1]MIVEGEC, Univ. Montpellier, IRD, CNRS, Montpellier, 34090, France; david.roiz@ird.fr (D.R.) ;
[2]LATHEMA, Instituto Oswaldo Cruz, FIOCRUZ, Rio de Janeiro-RJ, 4364, Brazil; lourenco@ioc.fiocruz.br (R.L.O.)
[*]Correspondence: tayssadnz@gmail.com (T.P.S.); christophe.paupy@ird.fr (C.P.)

Received: date; Accepted: date; Published: date

Table S1. List of the 16 articles found by searching Google Scholar to characterize the types of natural breeding sites exploited by *Ae. albopictus.*

**References**
Gomes, A.; Forattini, O.; Kakitani, I.; Marques, G.; Marques, C.; Marucci, D.; Brito, M. Microhabitats de Aedes albopictus (Skuse) na região do Vale do Paraíba, Estado de São Paulo, Brasil. *Rev. Saúde Pública S. Paulo* **1992**, *26*, 108–118.
Forattini, O.P.; Marques, G.R.A.M.; Kakitani, I.; De Brito, M.; Sallum, M.A.M. Significado epidemiológico dos criadouros de Aedes albopictus em bromélias. *Rev. Saude Publica* **1998**, *32*, 186–188.
Delatte, H.; Toty, C.; Boyer, S.; Bouetard, A.; Bastien, F.; Fontenille, D. Evidence of Habitat Structuring Aedes albopictus Populations in Réunion Island. *PLoS Negl. Trop. Dis.* **2013**, *7*, e2111.
Marquetti, M.; Bisset, J.; Leyva, M.; Garcia, A.; Rodriguez, M. Comportamiento estacional y temporal de Aedes aegypti y Aedes albopictus en La Habana, Cuba. **2008**, *60*, 62–67.
Li, Y.; Kamara, F.; Zhou, G.; Puthiyakunnon, S.; Li, C.; Liu, Y.; Zhou, Y.; Yao, L.; Yan, G.; Chen, X.G. Urbanization Increases Aedes albopictus Larval Habitats and Accelerates Mosquito Development and Survivorship. *PLoS Negl. Trop. Dis.* **2014**, *8*, e3301.
Hiscox, A.; Kaye, A.; Vongphayloth, K.; Banks, I.; Piffer, M.; Khammanithong, P.; Sananikhom, P.; Kaul, S.; Hill, N.; Lindsay, S.W.; et al. Risk factors for the presence of Aedes aegypti and Aedes albopictus in domestic water-holding containers in areas impacted by the Nam Theun 2 hydroelectric project, Laos. *Am. J. Trop. Med. Hyg.* **2013**, *88*, 1070–1078.
Marque Marquetti, M.; Bisset, J.; Leyva, M.; Garcia, A.; Rodriguez, M. Comportamiento estacional y temporal de Aedes aegypti y Aedes albopictus en La Habana, Cuba. **2008**, *60*, 62–67.
Amerasinghe, F.P.; Alagoda, T.S.B. Mosquito oviposition in bamboo traps, with special reference to Aedes albopictus, Aedes novalbopictus and Armigeres subalbatus. *Insect Sci. Applic.* **1984**, *5*, 493–500.
Gilotra, S.K.; Rozeboom, L.E.; Bhattacharya, N.C. Observations on possible competitive displacement between populations of Aedes aegypti Linnaeus and Aedes albopictus Skuse in Calcutta. *Bull. World Health Organ.* **1967**, *37*, 437–446.
Kamgang, B.; Ngoagouni, C.; Manirakiza, A.; Nakouné, E.; Paupy, C.; Kazanji, M. Temporal Patterns of Abundance of Aedes aegypti and Aedes albopictus (Diptera: Culicidae) and Mitochondrial DNA Analysis of Ae. albopictus in the Central African Republic. *PLoS Negl. Trop. Dis.* **2013**, *7*, e2590.
Forattini, O.P.; Brito, M. de B. Brief Communication an Unusual Grround Larval Habitat of Aedes albopictus. *Rev. Inst. Med. Trop. Sao Paulo* **1998**, *40*, 121–122.
Simard, F.; Nchoutpouen, E.; Toto, J.C.; Fontenille, D. Geographic distribution and breeding site preference of Aedes albopictus and Aedes aegypti (Diptera: culicidae) in Cameroon, Central Africa. *J Med Entomol* **2005**, *42*, 726–731.
Sota, T.; Mogi, M.; Hayamizu, E. Seasonal distribution and habitat selection by Aedes albopictus and Aedes riversi (Diptera®: Culicidae) in Northern Kyushu, Japan. *J. Med. Entomol.* **1992**, *29*, 296–304.
Thavara, U.; Tawatsin, a; Chansang, C.; Kong-ngamsuk, W.; Paosriwong, S.; Boon-Long, J.; Rongsriyam, Y.; Komalamisra, N. Larval occurrence, oviposition behavior and biting activity of potential mosquito vectors of dengue on Samui Island, Thailand. *J. Vector Ecol.* **2001**, *26*, 172–180.
Brito, M. d.; Forattini, O.P. Produtividade de criadouros de Aedes aegypti no Vale do ParaIba, SP, Brasil. *Rev. saúde pública* **2004**, *38*, 209–215.
Chan, K.L.; Ho, B.C.; Chan, Y.C. Aedes aegypti (L.) and Aedes albopictus (Skuse) in Singapore City. 2. Larval habitats. *Bull. World Health Organ.* **1971**, *44*, 629–633.

Table S2. Typology and number of reported natural containers exploited by *Ae. albopictus* from articles found in PubMed.

Type of natural container	Number of reported breeding sites	References
Bamboo stumps	127	
	1	Delatte, H.; Dehecq, J.S.; Thiria, J.; Domerg, C.; Paupy, C.; Fontenille, D. Geographic Distribution and Developmental Sites of Aedes albopictus (Diptera: Culicidae) During a Chikungunya Epidemic Event. *Vector Control Southeast Asia* **2008**, *8*, 25–34.
	46	Dev, V. Dengue vectors in urban and suburban Assam, India: entomological observations. *WHO South-East Asia J. Public Heal.* **2014**, *3*, 5838.
	17	Edillo, F.E.; Roble, N.D.; Otero, N.D. The key breeding sites by pupal survey for dengue mosquito vectors, Aedes aegypti (Linnaeus) and Aedes albopictus (Skuse), in Guba, Cebu City, Philippines. *Southeast Asian J. Trop. Med. Public Health* **2012**, *43*, 1365–1374.
	12	Gilotra, S.K.; Rozeboom, L.E.; Bhattacharya, N.C. Observations on possible competitive displacement between populations of Aedes aegypti Linnaeus and Aedes albopictus Skuse in Calcutta. *Bull. World Health Organ.* **1967**, *37*, 437–446.
	21	Rozeboom, L.E.; Bridges, J.R. Relative population densities of Aedes albopictus and A. guamensis on Guam. *Bull. World Health Organ.* **1972**, *46*, 477–483.
	30	Sota, T.; Mogi, M.; Hayamizu, E. Seasonal distribution and habitat selection by Aedes albopictus and Aedes riversi (Diptera®: Culicidae) in Northern Kyushu, Japan. *J. Med. Entomol.* **1992**, *29*, 296–304.
Bromeliads	291	
	3	Forattini, O.P.; Brito, M. de B. Brief Communication an Unusual Grround Larval Habitat of Aedes albopictus. *Rev. Inst. Med. Trop. Sao Paulo* **1998**, *40*, 121–122.
	275	Marques, G.R.; Forattini, O.P. Aedes albopictus in soil bromeliads in Ilhabela, coastal area of Southeastern Brazil. *Rev Saude Publica* **2005**, *39*, 548–552.
	7	Mocellin, M.G.; Simões, T.C.; do Nascimento, T.F.S.; Teixeira, M.L.F.; Lounibos, L.P.; de Oliveira, R.L. Bromeliad-inhabiting mosquitoes in an urban botanical garden of dengue endemic Rio de Janeiro - Are bromeliads productive habitats for the invasive vectors Aedes aegypti and Aedes albopictus? *Mem. Inst. Oswaldo Cruz* **2009**, *104*, 1171–1176.
	5	Oliveira, V.C. de; Almeida Neto, L.C. de Ocorrência de Aedes aegypti e Aedes albopictus em bromélias cultivadas no Jardim Botânico Municipal de Bauru, São Paulo, Brasil. *Cad. Saude Publica* **2017**, *33*, e00071016.
	1	O'Meara, G.F.; Cutwa, M.M.; Evans, L.F. Bromeliad-inhabiting mosquitoes in south Florida: native and exotic plants differ in species composition. *J. vector Ecol.* **2003**, *28*, 37–46.
Cacao shells	3	
	3	Simard, F.; Nchoutpouen, E.; Toto, J.C.; Fontenille, D. Geographic distribution and breeding site preference of Aedes albopictus and Aedes aegypti (Diptera: culicidae) in Cameroon, Central Africa. *J Med Entomol* **2005**, *42*, 726–731.
Coconut shells	838	
	8	Bagny, L.; Delatte, H.; Elissa, N.; Quilici, S.; Fontenille, D.; Adhami, J.; Murati, N.; Adhami, J.; Reiter, P.; Beltrame, A.; et al. Aedes (Diptera: Culicidae) vectors of arboviruses in Mayotte (Indian Ocean): distribution area and larval habitats. *J. Med. Entomol.* **2009**, *46*, 198–207.
	283	Banerjee, S.; Aditya, G.; Saha, G.K. Household disposables as breeding habitats of dengue vectors: Linking wastes and public health. *Waste Manag.* **2013**, *33*, 233–239.

Table S2. Continued

Type of natural container	Number of reported breeding sites	References
Coconut shells	838	
	7	Edillo, F.E.; Roble, N.D.; Otero, N.D. The key breeding sites by pupal survey for dengue mosquito vectors, Aedes aegypti (Linnaeus) and Aedes albopictus (Skuse), in Guba, Cebu City, Philippines. *Southeast Asian J. Trop. Med. Public Health* **2012**, *43*, 1365–1374.
	144	Rao, B.B.; George, B. Breeding patterns of aedes stegomyia albopictus in periurban areas of Calicut, Kerala, India. *Southeast Asian J. Trop. Med. Public Health* **2010**, *41*, 536–540.
	36	Rozeboom, L.E.; Bridges, J.R. Relative population densities of Aedes albopictus and A. guamensis on Guam. *Bull. World Health Organ.* **1972**, *46*, 477–483.
	20	Shriram, A.N.; Sivan, A.; Sugunan, A.P. Spatial distribution of Aedes aegypti and Aedes albopictus in relation to geo-ecological features in South Andaman, Andaman and Nicobar Islands, India. *Bull. Entomol. Res.* **2018**, *108*, 166–174.
	1	Simard, F.; Nchoutpouen, E.; Toto, J.C.; Fontenille, D. Geographic distribution and breeding site preference of Aedes albopictus and Aedes aegypti (Diptera: culicidae) in Cameroon, Central Africa. *J Med Entomol* **2005**, *42*, 726–731.
	316	Thavara, U.; Tawatsin, a; Chansang, C.; Kong-ngamsuk, W.; Paosriwong, S.; Boon-Long, J.; Rongsriyam, Y.; Komalamisra, N. Larval occurrence, oviposition behavior and biting activity of potential mosquito vectors of dengue on Samui Island, Thailand. *J. Vector Ecol.* **2001**, *26*, 172–180.
	22	Vijayakumar, K.; Sudheesh Kumar, T.K.; Nujum, Z.T.; Umarul, F.; Kuriakose, A. A study on container breeding mosquitoes with special reference to Aedes (Stegomyia) aegypti and Aedes albopictus in Thiruvananthapuram district, India. *J. Vector Borne Dis.* **2014**, *51*, 27–32.
Dead cow horns	3	
	3	Simard, F.; Nchoutpouen, E.; Toto, J.C.; Fontenille, D. Geographic distribution and breeding site preference of Aedes albopictus and Aedes aegypti (Diptera: culicidae) in Cameroon, Central Africa. *J Med Entomol* **2005**, *42*, 726–731.
Dead leaves	5	
	1	Bagny, L.; Delatte, H.; Elissa, N.; Quilici, S.; Fontenille, D.; Adhami, J.; Murati, N.; Adhami, J.; Reiter, P.; Beltrame, A.; et al. Aedes (Diptera: Culicidae) vectors of arboviruses in Mayotte (Indian Ocean): distribution area and larval habitats. *J. Med. Entomol.* **2009**, *46*, 198–207.
	4	Simard, F.; Nchoutpouen, E.; Toto, J.C.; Fontenille, D. Geographic distribution and breeding site preference of Aedes albopictus and Aedes aegypti (Diptera: culicidae) in Cameroon, Central Africa. *J Med Entomol* **2005**, *42*, 726–731.
Ground cavity	1	
	1	Forattini, O.P.; Brito, M. de B. Brief Communication an Unusual Grround Larval Habitat of Aedes albopictus. *Rev. Inst. Med. Trop. Sao Paulo* **1998**, *40*, 121–122.
Hollow log	1	
	1	Rozeboom, L.E.; Bridges, J.R. Relative population densities of Aedes albopictus and A. guamensis on Guam. *Bull. World Health Organ.* **1972**, *46*, 477–483.
Leaf axils	16	
	1	Banerjee, S.; Aditya, G.; Saha, G.K. Household disposables as breeding habitats of dengue vectors: Linking wastes and public health. *Waste Manag.* **2013**, *33*, 233–239.
	4	Delatte, H.; Dehecq, J.S.; Thiria, J.; Domerg, C.; Paupy, C.; Fontenille, D. Geographic Distribution and Developmental Sites of Aedes albopictus (Diptera: Culicidae) During a Chikungunya Epidemic Event. *Vector Control Southeast Asia* **2008**, *8*, 25–34.

Table S2. Continued

Type of natural container	Number of reported breeding sites	References
Leaf axils	16	
	6	Dev, V. Dengue vectors in urban and suburban Assam, India: entomological observations. *WHO South-East Asia J. Public Heal.* **2014**, *3*, 5838.
	1	Edillo, F.E.; Roble, N.D.; Otero, N.D. The key breeding sites by pupal survey for dengue mosquito vectors, Aedes aegypti (Linnaeus) and Aedes albopictus (Skuse), in Guba, Cebu City, Philippines. *Southeast Asian J. Trop. Med. Public Health* **2012**, *43*, 1365–1374.
	1	Kamgang, B.; Ngoagouni, C.; Manirakiza, A.; Nakouné, E.; Paupy, C.; Kazanji, M. Temporal Patterns of Abundance of Aedes aegypti and Aedes albopictus (Diptera: Culicidae) and Mitochondrial DNA Analysis of Ae. albopictus in the Central African Republic. *PLoS Negl. Trop. Dis.* **2013**, *7*, e2590.
	3	Shriram, A.N.; Sivan, A.; Sugunan, A.P. Spatial distribution of Aedes aegypti and Aedes albopictus in relation to geo-ecological features in South Andaman, Andaman and Nicobar Islands, India. *Bull. Entomol. Res.* **2018**, *108*, 166–174.
Palm bracts	7	
	7	Rozeboom, L.E.; Bridges, J.R. Relative population densities of Aedes albopictus and A. guamensis on Guam. *Bull. World Health Organ.* **1972**, *46*, 477–483.
Palm leaves	55	
	55	Delatte, H.; Dehecq, J.S.; Thiria, J.; Domerg, C.; Paupy, C.; Fontenille, D. Geographic Distribution and Developmental Sites of Aedes albopictus (Diptera: Culicidae) During a Chikungunya Epidemic Event. *Vector Control Southeast Asia* **2008**, *8*, 25–34.
Puddle	1	
	1	Marquetti, M.; Bisset, J.; Leyva, M.; Garcia, A.; Rodriguez, M. Comportamiento estacional y temporal de Aedes aegypti y Aedes albopictus en La Habana, Cuba. **2008**, *60*, 62–67.
Rock holes	49	
	1	Bagny, L.; Delatte, H.; Elissa, N.; Quilici, S.; Fontenille, D.; Adhami, J.; Murati, N.; Adhami, J.; Reiter, P.; Beltrame, A.; et al. Aedes (Diptera: Culicidae) vectors of arboviruses in Mayotte (Indian Ocean): distribution area and larval habitats. *J. Med. Entomol.* **2009**, *46*, 198–207.
	33	Delatte, H.; Dehecq, J.S.; Thiria, J.; Domerg, C.; Paupy, C.; Fontenille, D. Geographic Distribution and Developmental Sites of Aedes albopictus (Diptera: Culicidae) During a Chikungunya Epidemic Event. *Vector Control Southeast Asia* **2008**, *8*, 25–34.
	8	O′Meara, G.F.; Evans Jr., L.F.; Womack, M.L. Colonization of rock holes by Aedes albopictus in the southeastern United States. *J Am Mosq Control Assoc* **1997**, *13*, 270–274.
	3	Pena, C.J.; Gonzalvez, G.; Chadee, D.D. Seasonal prevalence and container preferences of Aedes albopictus in Santo Domingo City, Dominican Republic. *J. Vector Ecol.* **2003**, *28*, 208–212.
	1	Rozeboom, L.E.; Bridges, J.R. Relative population densities of Aedes albopictus and A. guamensis on Guam. *Bull. World Health Organ.* **1972**, *46*, 477–483.
	1	Shriram, A.N.; Sivan, A.; Sugunan, A.P. Spatial distribution of Aedes aegypti and Aedes albopictus in relation to geo-ecological features in South Andaman, Andaman and Nicobar Islands, India. *Bull. Entomol. Res.* **2018**, *108*, 166–174.
	2	Simard, F.; Nchoutpouen, E.; Toto, J.C.; Fontenille, D. Geographic distribution and breeding site preference of Aedes albopictus and Aedes aegypti (Diptera: culicidae) in Cameroon, Central Africa. *J Med Entomol* **2005**, *42*, 726–731.

Table S2. Continued

Type of natural container	Number of reported breeding sites	References
Snail shells	10	
	9	Rozeboom, L.E.; Bridges, J.R. Relative population densities of Aedes albopictus and A. guamensis on Guam. Bull. World Health Organ. 1972, 46, 477–483.
	1	Simard, F.; Nchoutpouen, E.; Toto, J.C.; Fontenille, D. Geographic distribution and breeding site preference of Aedes albopictus and Aedes aegypti (Diptera: culicidae) in Cameroon, Central Africa. J Med Entomol 2005, 42, 726–731.
Tree holes	125	
	1	Bagny, L.; Delatte, H.; Elissa, N.; Quilici, S.; Fontenille, D.; Adhami, J.; Murati, N.; Adhami, J.; Reiter, P.; Beltrame, A.; et al. Aedes (Diptera: Culicidae) vectors of arboviruses in Mayotte (Indian Ocean): distribution area and larval habitats. J. Med. Entomol. 2009, 46, 198–207.
	32	Gomes, A.; Forattini, O.; Kakitani, I.; Marques, G.; Marques, C.; Marucci, D.; Brito, M. Microhabitats de Aedes albopictus (Skuse) na região do Vale do Paraíba, Estado de São Paulo, Brasil. Rev. Saúde Pública S. Paulo 1992, 26, 108–118.
	1	Delatte, H.; Dehecq, J.S.; Thiria, J.; Domerg, C.; Paupy, C.; Fontenille, D. Geographic Distribution and Developmental Sites of Aedes albopictus (Diptera: Culicidae) During a Chikungunya Epidemic Event. Vector Control Southeast Asia 2008, 8, 25–34.
	12	Müller, G.C.; Kravchenko, V.D.; Junnila, A.; Schlein, Y. Tree-hole breeding mosquitoes in Israel. J. Vector Ecol. 2012, 37, 102–109.
	5	O'Meara, G.F.; Evans Jr., L.F.; Womack, M.L. Colonization of rock holes by Aedes albopictus in the southeastern United States. J Am Mosq Control Assoc 1997, 13, 270–274.
	13	Rozeboom, L.E.; Bridges, J.R. Relative population densities of Aedes albopictus and A. guamensis on Guam. Bull. World Health Organ. 1972, 46, 477–483.
	26	Shriram, A.N.; Sivan, A.; Sugunan, A.P. Spatial distribution of Aedes aegypti and Aedes albopictus in relation to geo-ecological features in South Andaman, Andaman and Nicobar Islands, India. Bull. Entomol. Res. 2018, 108, 166–174.
	2	Simard, F.; Nchoutpouen, E.; Toto, J.C.; Fontenille, D. Geographic distribution and breeding site preference of Aedes albopictus and Aedes aegypti (Diptera: culicidae) in Cameroon, Central Africa. J Med Entomol 2005, 42, 726–731.
	8	Sivan, A.; Shriram, A.N.; Sugunan, A.P.; Anwesh, M.; Muruganandam, N.; Kartik, C.; Vijayachari, P. Natural transmission of dengue virus serotype 3 by Aedes albopictus (Skuse) during an outbreak in Havelock Island: Entomological characteristics. Acta Trop. 2016, 156, 122–129.
	16	Sota, T.; Mogi, M.; Hayamizu, E. Seasonal distribution and habitat selection by Aedes albopictus and Aedes riversi (Diptera®: Culicidae) in Northern Kyushu, Japan. J. Med. Entomol. 1992, 29, 296–304.
	9	Urbinatti, P.R.; Menezes, R.M.T.; Natal, D. Sazonalidade de Aedes albopictus em área protegida na cidade de São Paulo , Brasil. Rev. Saude Publica 2007, 41, 478–481.

Table S3: List of references used to analyse the host feeding preferences of *Aedes albopictus*.

**References**
Almeida, P.; Baptista, S.; Sousa, C.; Novo, T.; Ramos, H.; Panella, N.; Godsey, M.; Simões, M.J.; Anselmo, M.L.; Komar, N.; et al. Bioecology and Vectorial Capacity of Aedes albopictus (Diptera: Culicidae) in Macao, China, in Relation to Dengue Virus Transmission. *J. Med. Entomol.* **2005**, *42*, 419–428.
Colless, D.H. Notes on the culicine mosquitoes of singapore. *Ann. Trop. Med. Parasitol.* **1959**, *51*, 87–101.
Dennett, J. a; Bala, A.; Wuithiranyagool, T.; Randle, Y.; Sargent, C.B.; Guzman, H.; Siirin, M.; Hassan, H.K.; Reyna-Nava, M.; Unnasch, T.R.; et al. Associations between two mosquito populations and West Nile virus in Harris County, Texas, 2003-06. *J. Am. Mosq. Control Assoc.* **2007**, *23*, 264–275.
Pereira dos Santos, T.; Roiz, D.; de Abreu, F.; Luz Bessa, S.; Santa Lucia, M.; Jiolle, D.; Neves, N.; Simard, F.; Lourenço De Oliveira, R.; Paupy, C. Potential of Aedes albopictus as a bridge vector for zoonotic pathogens at the urban-forest interface of Brazil. *Emerg. Microbes Infect.* **2018**, *7*, 191.
Faraji, A.; Egizi, A.; Fonseca, D.M.; Unlu, I.; Crepeau, T.; Healy, S.P.; Gaugler, R. Comparative Host Feeding Patterns of the Asian Tiger Mosquito, Aedes albopictus, in Urban and Suburban Northeastern USA and Implications for Disease Transmission. *PLoS Negl. Trop. Dis.* **2014**, *8*, e3037.
Gingrich, J.B.; Williams, G.M. Host-feeding patterns of suspected West Nile virus mosquito vectors in Delaware, 2001-2002. *J. Am. Mosq. Control Assoc.* **2005**, *21*, 194–200.
Gomes, A.; Silva, N.; Marques, G.; Brito, M. Host-feeding patterns of potential human disease vectors in the Paraiba Valley Region, State of Sao Paulo, Brazil 2002, 28: 74–78.
Kamgang, B.; Nchoutpouen, E.; Simard, F.; Paupy, C. Notes on the blood-feeding behavior of Aedes albopictus (Diptera: Culicidae) in Cameroon. *Parasit. Vectors* **2012**, *5*, 57.
Kek, R.; Hapuarachchi, H.C.; Chung, C.-Y.; Humaidi, M. Bin; Razak, M.A.B.A.; Chiang, S.; Lee, C.; Tan, C.-H.; Yap, G.; Chong, C.-S.; et al. Feeding Host Range of *Aedes albopictus* (Diptera: Culicidae) Demonstrates Its Opportunistic Host-Seeking Behavior in Rural Singapore. *J. Med. Entomol.* **2014**, *51*, 880–884.
Kim, K.S.; Tsuda, Y.; Yamada, A. Bloodmeal Identification and Detection of Avian Malaria Parasite From Mosquitoes (Diptera: Culicidae) Inhabiting Coastal Areas of Tokyo Bay, Japan. *J. Med. Entomol.* **2009**, *46*, 1230–1234.
Kim, H.; Yu, H. mi; Lim, H.W.; Yang, S.C.; Roh, J.Y.; Chang, K.S.; Shin, E.H.; Ju, Y.R.; Lee, W.G. Host-feeding pattern and dengue virus detection of Aedes albopictus (Diptera: Culicidae) captured in an urban park in Korea. *J. Asia. Pac. Entomol.* **2017**, *20*, 809–813.
Muñoz, J.; Eritja, R.; Alcaide, M.; Montalvo, T.; Soriguer, R.C.; Figuerola, J. Host-Feeding Patterns of Native Culex pipiens and Invasive Aedes albopictus Mosquitoes (Diptera: Culicidae) in Urban Zones From Barcelona, Spain. *J. Med. Entomol.* **2011**, *48*, 956–960.
Niebylski, M.L.; Savage, H.M.; Nasci, R.S.; Craig, G.B. Blood hosts of Aedes albopictus in the United States. *J. Am. Mosq. Control Assoc.* **1994**, *10*, 447–450.
Ponlawat, A.; Harrington, L.C. Blood Feeding Patterns of Aedes aegypti and Aedes albopictus in Thailand. *J. Med. Entomol.* **2005**, *42*, 844–849.
Richards, S.L.; Ponnusamy, L.; Unnasch, T.R.; Hassan, H.K.; Apperson, C.S. Host-Feeding Patterns of Aedes albopictus (Diptera: Culicidae) in Relation to Availability of Human and Domestic Animals in Suburban Landscapes of Central North Carolina. *J. Med. Entomol.* **2006**, *43*, 543–551.
Savage, H.; Niebylski, M.; Smith, G.; Mitchell, C.; Craig, G. Host-feeding patterns of Aedes albopictus (Diptera: Culicidae) at a temperate North American site. *J. Med. Entomol.* **1993**, *30*, 27–34.
Sawabe, K.; Isawa, H.; Hoshino, K.; Sasaki, T.; Roychoudhury, S.; Higa, Y.; Kasai, S.; Tsuda, Y.; Nishiumi, I.; Hisai, N.; et al. Host-Feeding Habits of Culex pipiens and Aedes albopictus (Diptera: Culicidae) Collected at the Urban and Suburban Residential Areas of Japan. *J. Med. Entomol.* **2010**, *47*, 442–450.
Sivan, A.; Shriram, A.; Sunish, I.; Vidhya, P. Host-feeding pattern of Aedes aegypti and Aedes albopictus (Diptera: Culicidae) in heterogeneous landscapes of South Andaman, Andaman and Nicobar Islands, India. *Parasitol. Res.* **2015**, *114*, 3539–3546.
Tandon, N.; Ray, S. Host feeding pattern of Aedes aegypti and Aedes albopictus in Kolkata, India. *Denuge Bull.* **2000**, *24*, 117–120.
Tempelis, C.H.; Hayes, R.O.; Hess, A.D.; Reeves, W.C. Blood-feeding habits of four species of mosquito found in Hawaii. *Am. J. Trop. Med. Hyg.* **1970**, *19*, 335–341.
Valerio, L.; Francesca, M.; Bongiorno, G.; Facchinelli, L.; Pombi, M.; Caputo, B.; Maroli, M.; Torre, A. Della Host-Feeding Patterns of Aedes albopictus. **2010**, *10*, 291–294.
Egizi, A.; Healy, S.P.; Fonseca, D.M. Rapid blood meal scoring in anthropophilic Aedes albopictus and application of PCR blocking to avoid pseudogenes. *Infect. Genet. Evol.* **2013**, *16*, 122–128.

Table S4: List of references that reported infection, infections rate, dissemination rate, dissemination efficiency, transmissions rate, transmission efficiency or transovarial transmission in Ae. albopictus for the indicated arboviruses.

Virus Name	References
Arumowot	Tesh, R.B. Multiplication Phlebotomus phlebotomus fever group arboviruses in mosquitoes after intrathoracic inoculation. *J. Med. Entomol.* **1975**, *12*, 1–4.
BeAn 100049 (Urucuri)	Tesh, R.B. Multiplication Phlebotomus phlebotomus fever group arboviruses in mosquitoes after intrathoracic inoculation. *J. Med. Entomol.* **1975**, *12*, 1–4.
Bujaru	Tesh, R.B. Multiplication Phlebotomus phlebotomus fever group arboviruses in mosquitoes after intrathoracic inoculation. *J. Med. Entomol.* **1975**, *12*, 1–4.
Bussuquara	Tesh, R.B.; Shroyer, D.A. The mechanism of arbovirus transovarial transmission in mosquitoes: San Angelo virus in Aedes albopictus. *Am. J. Trop. Med. Hyg.* **1980**, *29*, 1394–1404.
Cache Valley	Armstrong, P.M.; Anderson, J.F.; Farajollahi, A.; Healy, S.P.; Unlu, I.; Crepeau, T.N.; Gaugler, R.; Fonseca, D.M.; Andreadis, T.G. Isolations of Cache Valley virus from Aedes albopictus (Diptera: Culicidae) in New Jersey and evaluation of its role as a regional arbovirus vector. *J. Med. Entomol.* **2013**, *50*, 1310–1314.
Chandipura	Ramachandra, R.; Sing, K.; Dhanda, V.; Bhattacharya, N. Experimental transmission of Chandipyura Virus by mosquitoes. *Ind. Jour. Med. Res.* **1967**, *55*, 1306–1310.
Chilibre	Tesh, R.B. Multiplication Phlebotomus phlebotomus fever group arboviruses in mosquitoes after intrathoracic inoculation. *J. Med. Entomol.* **1975**, *12*, 1–4.
Eastern equine encephalomyelitis	Mitchell, C.J.; McLean, R.G.; Nasci, R.S.; Crans, W.J.; Smith, G.C.; Caccamise, D.F. Susceptibility parameters of Aedes albopictus to per oral infection with eastern equine encephalitis virus. *J Med Entomol* **1993**, *30*, 233–235.<br>Moncayo, a C.; Edman, J.D.; Turell, M.J. Effect of eastern equine encephalomyelitis virus on the survival of Aedes albopictus, Anopheles quadrimaculatus, and Coquillettidia perturbans (Diptera: Culicidae). *J. Med. Entomol.* **2000**, *37*, 701–706.<br>Scott, T.W.; Lorenz, L.H.; Weaver, S.C. Susceptibility of Aedes albopictus to infection with eastern equine encephalomyelitis virus. *J. Am. Mosq. Control Assoc.* **1990**, *6*, 274–278.<br>Turell, M.; Beaman, J.; Neely, G. Experimental transmission of Eastern equine encephalitis virus by strains of Aedes albopictus and A. taeniorhynchus (Diptera: Culicidae). *J. Med. Entomol.* **1994**, *31*, 287–290.
Getah	Takashima, I.; Hashimoto, N. Getah virus in several species of mosquitoes. *Trans. R. Soc. Trop. Med. Hyg.* **1985**, *79*, 546–550.
Icoaraci	Tesh, R.B. Multiplication Phlebotomus phlebotomus fever group arboviruses in mosquitoes after intrathoracic inoculation. *J. Med. Entomol.* **1975**, *12*, 1–4.
Ilheus	Tesh, R.B. Experimental studies on the transovarial transmission of Kunjin and San Angelo viruses in mosquitoes. *Am. J. Trop. Med. Hyg.* **1980**, *29*, 657–666.
Itaporanga	Tesh, R.B. Multiplication Phlebotomus phlebotomus fever group arboviruses in mosquitoes after intrathoracic inoculation. *J. Med. Entomol.* **1975**, *12*, 1–4.
Jamestown Canyon	Grimstad, P.R. Recently Introduced Aedes albopictus in the United States: Potential vector of La Crosse Virus (Bunyaviridae: California Serogroup)1. **1989**, *5*, 422–427.
Japanese encephalitis	Rosen, L.; Tesh, R.B.; Lien, J.; John, H. Transovarial Transmission of Japanese Encephalitis Virus by Mosquitoes. *Am. Assoc. Adv. Sci.* **1978**, *199*, 909–911.<br>Tesh, R.B. Experimental studies on the transovarial transmission of Kunjin and San Angelo viruses in mosquitoes. *Am. J. Trop. Med. Hyg.* **1980**, *29*, 657–666.<br>Weng, M.H.; Lien, J.C.; Wang, Y.M.; Wu, H.L.; Chin, C. Susceptibility of three laboratory strains of Aedes albopictus (Diptera: Culicidae) to Japanese encephalitis virus from Taiwan. *J Med Entomol* **1997**, *34*, 745–747.<br>Wispelaere, M.; Desprès, P.; Choumet, V. European Aedes albopictus and Culex pipiens Are Competent Vectors for Japanese Encephalitis Virus. *PLoS Negl. Trop. Dis.* **2017**, *11*, e0005294.
Karimabad	Tesh, R.B. Multiplication Phlebotomus phlebotomus fever group arboviruses in mosquitoes after intrathoracic inoculation. *J. Med. Entomol.* **1975**, *12*, 1–4.
Keystone	Grimstad, P.R. Recently Introduced Aedes albopictus in the United States: Potential vector of La Crosse Virus (Bunyaviridae: California Serogroup)1. **1989**, *5*, 422–427.

Table S4. Continued

Virus Name	References
Keystone	Tesh, R.B. Experimental studies on the transovarial transmission of Kunjin and San Angelo viruses in mosquitoes. *Am. J. Trop. Med. Hyg.* **1980**, *29*, 657–666.
kokobera	Tesh, R.B. Experimental studies on the transovarial transmission of Kunjin and San Angelo viruses in mosquitoes. *Am. J. Trop. Med. Hyg.* **1980**, *29*, 657–666.
Kunjin	Tesh, R.B. Experimental studies on the transovarial transmission of Kunjin and San Angelo viruses in mosquitoes. *Am. J. Trop. Med. Hyg.* **1980**, *29*, 657–666.
La Crosse	Cully Jr., J.F.; Streit, T.G.; Heard, P.B. Transmission of La Crosse virus by four strains of Aedes albopictus to and from the eastern chipmunk (Tamias striatus). *J Am Mosq Control Assoc* **1992**, *8*, 237–240.<br>Grimstad, P.R. Recently Introduced Aedes albopictus in the United States: Potential vector of La Crosse Virus (Bunyaviridae: California Serogroup)1. **1989**, *5*, 422–427.<br>Hughes, M.T.; Gonzalez, J.A.; Reagan, K.L.; Carol, D.; Beaty, B.J.; Beaty, B.J. Comparative Potential of Aedes triseriatus , Aedes albopictus , and Aedes aegypti (Diptera®: Culicidae) to Transovarially Transmit La Crosse Virus Comparative Potential of Aedes triseriatus , Aedes albopictus , and Aedes aegypti (Diptera®: Culicidae). *Entomol. Soc. Am.* **2006**, *43*, 757–761.<br>Lambert, A.J.; Blair, C.D.; D'Anton, M.; Ewing, W.; Harborth, M.; Seiferth, R.; Xiang, J.; Lanciotti, R.S. La Crosse Virus in Aedes albopictus Mosquitoes, Texas, USA, 2009. *Emerg. Infect. Dis.* **2010**, *16*, 856–858.<br>Tesh, R.B. Experimental studies on the transovarial transmission of Kunjin and San Angelo viruses in mosquitoes. *Am. J. Trop. Med. Hyg.* **1980**, *29*, 657–666.
Mayaro	Smith, G.C.; Francy, D.B. Laboratory studies of a Brazilian strain of Aedes albopictus as a potential vector of Mayaro and Oropouche viruses. *J Am Mosq Control Assoc* **1991**, *7*, 89–93.
Oropuche	Smith, G.C.; Francy, D.B. Laboratory studies of a Brazilian strain of Aedes albopictus as a potential vector of Mayaro and Oropouche viruses. *J Am Mosq Control Assoc* **1991**, *7*, 89–93.
Orungo	Tomori, O.; Aitken, T.H. Orungo virus: transmission studies with Aedes albopictus and Aedes aegypti (Diptera: Culicidae). *J Med Entomol* **1978**, *14*, 523–526.
Pacui	Tesh, R.B. Multiplication Phlebotomus phlebotomus fever group arboviruses in mosquitoes after intrathoracic inoculation. *J. Med. Entomol.* **1975**, *12*, 1–4.
Potosi	Francy, D.; Krabatsos, N.; Wesson, C.; Moore, J.; Lazuick, J.; Niebylski, T.; Tsai, T.; Craig, J. A new Arbovirus from Aedes albopictus, an Asian mosquito Established in the United States. *Science (80-.).* **1990**, *250*, 1738–1740.<br>Heard, P.B.; Niebylski, M.L.; Francy, D.B. Transmission of a Newly Recognized Virus (Bunyaviridae , Bunyavirus) Isolated from Aedes albopictus (Diptera®: Culicidae) in Potosi , Missouri. *J. Med. Entomol.* **1991**, *28*, 601–605.<br>Mitchell, C.J.; Smith, G.C.; Miller, B.R. Vector Competence of Aedes Albopictus for a Newly Recognized Bunyavirus From Mosquitoes Collected in Potosi, Missouri1. *J. Am. Mosq. Control Assoc.* **1990**, *6*, 523–527.
Rift Valley	Brustolin, M.; Talavera, S.; Nunez, A.; SantamarÍa, C.; Rivas, R.; Pujol, N.; Valle, M.; Verdun, M.; Brun, A.; Pages, N.; et al. Rift Valley fever virus and European mosquitoes: vector competence of Culex pipiens and Stegomyia albopicta (= Aedes albopictus). *Med. Vet. Entomol.* **2017**, *31*, 365–372.<br>Turell, M.J.; Bailey, C.L.; Beaman, J.R. Vector competence of a Houston, Texas strain of Aedes albopictus for Rift Valley fever virus. *J. Am. Mosq. Control Assoc.* **1988**, *4*, 94–96.
Ross River	Mitchell, C.J.; Gubler, D.J. Vector competence of geographic strains of Aedes albopictus and Aedes polynesiensis and certain other Aedes (Stegomyia) mosquitoes for Ross River virus. *J Am Mosq Control Assoc* **1987**, *3*, 142–147.<br>Mitchell, C.J.; Miller, B.R.; Gubler, D.J. Vector competence of Aedes albopictus from Houston, Texas, for dengue serotypes 1 to 4, yellow fever and Ross River viruses. *J. Am. Mosq. Control Assoc.* **1987**, *3*, 460–465.
Salehabad	Tesh, R.B. Multiplication Phlebotomus phlebotomus fever group arboviruses in mosquitoes after intrathoracic inoculation. *J. Med. Entomol.* **1975**, *12*, 1–4.
San Angelo	Shroyer, D.A. Transovarial Maintenance of San Angelo Virus in Sequenctial Generations of Aedes albopictus. *Am J Trop Med Hyg.* **1986**, *35*, 408–417.<br>Tesh, R.B. Experimental studies on the transovarial transmission of Kunjin and San Angelo viruses in mosquitoes. *Am. J. Trop. Med. Hyg.* **1980**, *29*, 657–666.<br>Tesh, R.B. Multiplication Phlebotomus phlebotomus fever group arboviruses in mosquitoes after intrathoracic inoculation. *J. Med. Entomol.* **1975**, *12*, 1–4.

Table S4. Continued

Virus Name	References
Tensaw Virus	Mitchell, C.J. Vector competence of North and South American strains of Aedes albopictus for certain arboviruses: a review. *J. Am. Mosq. Control Assoc.* **1991**, *7*, 446–451.
Trivitatus Virus	Grimstad, P.R. Recently Introduced Aedes albopictus in the United States: Potential vector of La Crosse Virus (Bunyaviridae: California Serogroup)1. **1989**, *5*, 422–427.
Uganda S	Tesh, R.B. Experimental studies on the transovarial transmission of Kunjin and San Angelo viruses in mosquitoes. *Am. J. Trop. Med. Hyg.* **1980**, *29*, 657–666.
Usutu virus	Puggioli, A.; Bonilauri, P.; Calzolari, M.; Lelli, D.; Carrieri, M.; Urbanelli, S.; Pudar, D.; Bellini, R. Does Aedes albopictus (Diptera: Culicidae) play any role in Usutu virus transmission in Northern Italy? Experimental oral infection and field evidences. *Acta Trop.* **2017**, *172*, 192–196.
Venezuelan equine encephalitis	Beaman, J.R.; Turell, M.J. Transmission of Venezuelan Equine Encephalomyelitis Virus by Strains of Aedes albopictus (Diptera: Culicidae) Collected from North and South America. *J. Med. Entomol.* **1991**, *28*, 161–164. Fernandez, Z.; Moncayo, A.C.; Carrara, A.S.; Forattini, O.P.; Weaver, A.S.C. Vector Competence of Rural and Urban Strains of Aedes (Stegomyia) albopictus (Diptera: Culicidae) from São Paulo State, Brazil for IC, ID, and IF Subtypes of Venezuelan Equine Encephalitis Virus. *J. Med. Entomol* **2003**, *40*, 522–527. Turell, M.J.; Beaman, J.R. Experimental Transmission of Venezuelan Equine Encephalomyelitis Virus by a strain of Aedes albopictus (Diptera: Culicidae) from New Orleans, Lousiana. *J. Med. Entomol.* **1992**, *29*, 802–807.
West Nile	Baqar, S.; Hayes, C.G.; Murphy, J.R.; Watts, D.M. Vertical transmission of West Nile virus by Culex and Aedes species mosquitoes. *Am. J. Trop. Med. Hyg.* **1993**, *48*, 757–762. Brustolin, M.; Talavera, S.; Santamaría, C.; Rivas, R.; Pujol, N.; Aranda, C.; Marquès, E.; Valle, M.; Verdún, M.; Pagès, N.; et al. Culex pipiens and Stegomyia albopicta (=Aedes albopictus) populations as vectors for lineage 1 and 2 West Nile virus in Europe. *Med. Vet. Entomol.* **2016**, *30*, 166–173. Cupp, E.W.; Hassan, H.K.; Yue, X.; Oldland, W.K.; Lilley, B.M.; Unnasch, T.R. West Nile Virus Infection in Mosquitoes in the Mid-South USA, 2002–2005. *J. Med. Entomol.* **2007**, *44*, 117–125. Farajollahi, A.; Kesavaraju, B.; Price, D.C.; Williams, G.M.; Healy, S.P.; Gaugler, R.; Nelder, M.P. Field efficacy of BG-Sentinel and industry-standard traps for Aedes albopictus (Diptera: Culicidae) and West Nile virus surveillance. *J. Med. Entomol.* **2009**, *46*, 919–25. Fortuna, C.; Remoli, M.E.; Severini, F.; Di Luca, M.; Toma, L.; Fois, F.; Bucci, P.; Boccolini, D.; Romi, R.; Ciufolini, M.G. Evaluation of vector competence for West Nile virus in Italian Stegomyia albopicta (=Aedes albopictus) mosquitoes. *Med. Vet. Entomol.* **2015**, *29*, 430–433. Holick, J.; Kyle, A.; Ferraro, W.; Delaney, R.; Marta, I. Discovery of Aedes albopictus infected with west nile virus in Southeastern Pennsylvania. *J. Am. Mosq. Control Assoc. INC.* **2002**, *18*, 131. Philip, C.B.; Smadel, J.E. Transmission of West Nile Virus by Infected Aedes albopictus. C. *Proc Soc Exper Biol Med* **1943**, *53*, 49–50. Sardelis, M.R.; Turell, M.J.; Guinn, M.L.O.; Andre, R.G.; Roberts, D.R. Vector Competence of Three North American Strains of Aedes Albopictus for West Nile Virus1. *J. Am. Mosq. Control Assoc.* **2002**, *18*, 284–289. Tiawsirisup, S.; Platt, K.B.; Evans, R.B.; Rowley, W.A. A Comparision of West Nile Virus Transmission by Ochlerotatus trivittatus (COQ.), Culex pipiens (L.), and Aedes albopictus (Skuse). *Vector-Borne Zoonotic Dis.* **2005**, *5*, 40–47. Vanlandingham, D.L.; McGee, C.E.; Klinger, K.A.; Vessey, N.; Fredregillo, C.; Higgs, S. Short report: Relative susceptibilties of South Texas mosquitoes to infection with West Nile virus. *Am. J. Trop. Med. Hyg.* **2007**, *77*, 925–928.
Yellow fever	Amraoui, F.; Ayed, W. Ben; Madec, Y.; Faraj, C.; Himmi, O.; Btissam, A.; Sarih, M.; Failloux, A.B. Potential of aedes albopictus to cause the emergence of arboviruses in Morocco. *PLoS Negl. Trop. Dis.* **2019**, *13*, e0006997. Couto-Lima, Di.; Madec, Y.; Bersot, M.I.; Campos, S.S.; Motta, M.D.A.; Dos Santos, F.B.; Vazeille, M.; Da Costa Vasconcelos, P.F.; Lourenço-De-Oliveira, R.; Failloux, A.B. Potential risk of re-emergence of urban transmission of Yellow Fever virus in Brazil facilitated by competent Aedes populations. *Sci. Rep.* **2017**, *7*, 4848. Johnson, B.; Chambers, T.; Grabtree, M.; Filippis, A.; Vilarinhos, P.; Resende, M.; Marcoris, L.; Miller, B. Vector competence of Brazilian Aedes aegypti and Ae . abopictus for a Brazilian yellow fever virus isolate *. *Trans. R. Soc. Trop. Med. Hyg.* **2002**, *96*, 611–613.

Table S4. Continued

Virus Name	References
Yellow fever	Lourenço de Oliveira, R.; Vazeille, M.; Maria, A.N.A.; Filippis, B.D.E.; Failloux, A. Large genetic differentiation and low variation in vector competence for dengue and yellow fever viruses of Aedes albopictus from Brazil, the United States, and the Cayman Islands. Am. J. Trop. Med. Hyg. 2003, 69, 105–114.
	Miller, B.R.; Mitchell, C.J.; Ballinger, M.E. Replication, tissue tropisms and transmission of yellow fever virus in Aedes albopictus. Trans. R. Soc. Trop. Med. Hyg. 1989, 83, 252–255.
	Mitchell, C.J.; Miller, B.R.; Gubler, D.J. Vector competence of Aedes albopictus from Houston, Texas, for dengue serotypes 1 to 4, yellow fever and Ross River viruses. J. Am. Mosq. Control Assoc. 1987, 3, 460–465.

Table S5: Natural detection or isolation of arboviruses in *Ae. albopictus* from field-collected mosquitoes.

Virus Name	Detection	Isolation	References
CVV	-	+	Armstrong, P.M.; Anderson, J.F.; Farajollahi, A.; Healy, S.P.; Unlu, I.; Crepeau, T.N.; Gaugler, R.; Fonseca, D.M.; Andreadis, T.G. Isolations of Cache Valley virus from Aedes albopictus (Diptera: Culicidae) in New Jersey and evaluation of its role as a regional arbovirus vector. *J. Med. Entomol.* **2013**, *50*, 1310–1314.
	-	+	Mitchell, C.J.; Haramis, L.D.; Karabatsos, N.; Smith, G.C.; Starwalt, V.J. Isolation of La Crosse, Cache Valley, and Potosi Viruses from Aedes Mosquitoes (Diptera: Culicidae) Collected at Used-Tire Sites in Illinois during 1994-1995. *J. Med. Entomol.* **1998**, *35*, 573–577.
EEEV	-	+	Mitchell, C.J.; Niebylski, M.L.; Smith, G.C.; Karabatsos, N.; Martin, D.; Mutebi, J.P.; Craig, G.B.J.; Mahler, M.J. Isolation of eastern equine encephalitis virus from Aedes albopictus in Florida. *Science* **1992**, *257*, 526–527.
KEYV	-	+	Mitchell, C.J.; Niebylski, M.L.; Smith, G.C.; Karabatsos, N.; Martin, D.; Mutebi, J.P.; Craig, G.B.J.; Mahler, M.J. Isolation of eastern equine encephalitis virus from Aedes albopictus in Florida. *Science* **1992**, *257*, 526–527.
LACV	+	-	Westby, K.M.; Fritzen, C.; Paulsen, D.; Poindexter, S.; Moncayo, A.C. La Crosse Encephalitis Virus Infection in Field-Collected *Aedes albopictus* , *Aedes japonicus* , and *Aedes triseriatus* in Tennessee. *J. Am. Mosq. Control Assoc.* **2015**, *31*, 233–241.
	-	+	Gerhardt, R.R.; Gottfried, K.L.; Apperson, C.S.; Davis, B.S.; Erwin, P.C.; Smith, A.B.; Panella, N.A.; Powell, E.E.; Nasci, R.S. First isolation of La Crosse virus from naturally infected Aedes albopictus. *Emerg. Infect. Dis.* **2001**, *7*, 807–811.
	-	+	Lambert, A.J.; Blair, C.D.; D'Anton, M.; Ewing, W.; Harborth, M.; Seiferth, R.; Xiang, J.; Lanciotti, R.S. La Crosse Virus in Aedes albopictus Mosquitoes, Texas, USA, 2009. *Emerg. Infect. Dis.* **2010**, *16*, 856–858.
	-	+	Mitchell, C.J.; Haramis, L.D.; Karabatsos, N.; Smith, G.C.; Starwalt, V.J. Isolation of La Crosse, Cache Valley, and Potosi Viruses from Aedes Mosquitoes (Diptera: Culicidae) Collected at Used-Tire Sites in Illinois during 1994-1995. *J. Med. Entomol.* **1998**, *35*, 573–577.
POTV	-	+	Francy, D.; Krabatsos, N.; Wesson, C.; Moore, J.; Lazuick, J.; Niebylski, T.; Tsai, T.; Craig, J. A new Arbovirus from Aedes albopictus, an Asian mosquito Established in the United States. *Science (80-.).* **1990**, *250*, 1738–1740.
	-	+	Harrison, B.A.; Mitchell, C.J.; Apperson, C.S.; Smith, G.C.; Karabatsos, N.; Engber, B.R.; Newton, N.H. Isolation of potosi virus from Aedes albopictus in North Carolina. *J Am Mosq Control Assoc* **1995**, *11*, 225–229.
	-	+	Mitchell, C.J.; Haramis, L.D.; Karabatsos, N.; Smith, G.C.; Starwalt, V.J. Isolation of La Crosse, Cache Valley, and Potosi Viruses from Aedes Mosquitoes (Diptera: Culicidae) Collected at Used-Tire Sites in Illinois during 1994-1995. *J. Med. Entomol.* **1998**, *35*, 573–577.
TENV	-	+	Mitchell, C.J.; Niebylski, M.L.; Smith, G.C.; Karabatsos, N.; Martin, D.; Mutebi, J.P.; Craig, G.B.J.; Mahler, M.J. Isolation of eastern equine encephalitis virus from Aedes albopictus in Florida. *Science* **1992**, *257*, 526–527.
USUV	+	-	Puggioli, A.; Bonilauri, P.; Calzolari, M.; Lelli, D.; Carrieri, M.; Urbanelli, S.; Pudar, D.; Bellini, R. Does Aedes albopictus (Diptera: Culicidae) play any role in Usutu virus transmission in Northern Italy? Experimental oral infection and field evidences. *Acta Trop.* **2017**, *172*, 192–196.
WNV	+	-	Cupp, E.W.; Hassan, H.K.; Yue, X.; Oldland, W.K.; Lilley, B.M.; Unnasch, T.R. West Nile Virus Infection in Mosquitoes in the Mid-South USA, 2002–2005. *J. Med. Entomol.* **2007**, *44*, 117–125.
	+	-	Farajollahi, A.; Kesavaraju, B.; Price, D.C.; Williams, G.M.; Healy, S.P.; Gaugler, R.; Nelder, M.P. Field efficacy of BG-Sentinel and industry-standard traps for Aedes albopictus (Diptera: Culicidae) and West Nile virus surveillance. *J. Med. Entomol.* **2009**, *46*, 919–25.
	+	-	Holick, J.; Kyle, A.; Ferraro, W.; Delaney, R.; Marta, I. Discovery of Aedes albopictus infected with west nile virus in Southeastern Pennsylvania. *J. Am. Mosq. Control Assoc. INC.* **2002**, *18*, 131.

CCV, Cache Valley virus; EEEV, Eastern Equine Encephalomyelitis virus; KEYV, Keystone virus; LACV, La Crosse virus; POTV, Potosi virus; TENV, Tensaw virus; USUV, Usutu vírus; WNV, West Nile virus.

Table S6. Geographic distribution, vertebrate hosts and potential vectors of arboviruses isolated or tested for vector competence in *Ae. albopictus*. Information on hosts and virus isolation from field-collected mosquitoes are from the arbocat database (https://wwwn.cdc.gov/arbocat). Arbovirus isolated (+) or not (-) from *Ae. albopictus*.

Virus	Virus Family	Virus Genus	Geographic Distribution	Natural Host Family	Arthropod Isolation
Cache Valley(+)	Peribunyaviridae	Orthobunyavirus	Canada<br>USA<br>Jamaica	Atelidae<br>Canidae<br>Cervidae<br>Equidae<br>Humans<br>Procyonidae<br>Other unidentified animals	*Aedes sollicitans*<br>*Aedes taeniorhynchus*<br>*Anopheles crucians*<br>*Anopheles grabhamii*<br>*Anopheles quadrimaculatus*<br>*Culiseta inornata*<br>*Psorophora confinnis*
Eastern equine encephalomyelitis(+)	Togaviridae	Alphavirus	Argentina<br>Brazil<br>Colombia<br>Trinidad<br>Dominican<br>Guyana<br>Panama<br>Surinam<br>Eastern Canada<br>USA<br>Czechoslovakia<br>Thailand<br>Philippine Islands<br>Czechoslovakia	Bovidae<br>Canidae<br>Cricetidae<br>Didelphidae<br>Echimyidae<br>Equidae<br>Humans<br>Muridae<br>Phasianidae<br>Other unidentified animals	*Aedes atlanticus*<br>*Aedes fulvus pallens*<br>*Aedes mitchellae*<br>*Aedes sollicitans*<br>*Aedes sticticus*<br>*Aedes taeniorhynchus*<br>*Aedes vexans*<br>*Anopheles crucians*<br>*Coquillettidia pertubans*<br>*Culex nigripalpus*<br>*Culex quinquefasciatus*<br>*Culex restuans*<br>*Culex salinarius*<br>*Culex taeniopus*<br>*Culiseta melanura*<br>*Dermanyssus gallinae*<br>*Eomenocanthus stramineus*<br>*Eusimulium johannseni*<br>*Simulium meridionale*
Keystone(+)	Peribunyaviridae	Orthobunyavirus	USA	Cervidae<br>Cricetidae<br>Other unidentified animals	*Aedes atlanticus*<br>*Aedes aurifer*<br>*Aedes infirmatus*<br>*Aedes taeniorhynchus*<br>*Anopheles crucians*<br>*Culex nigripalpus*
La Crosse(+)	Peribunyaviridae	Orthobunyavirus	USA	Humans<br>Sciuridae	Unknown
Potosi(+)	Peribunyaviridae	Orthobunyavirus	USA	Unknown	Unknown
Tensaw(+)	Peribunyaviridae	Orthobunyavirus	USA	Bovidae<br>Bovidae<br>Canidae<br>Cricetidae<br>Humans<br>Leporidae<br>Phasianidae<br>Procyonidae<br>Other unidentified animals	*Aedes atlanticus*<br>*Aedes atlanticus*<br>*Aedes infirmatus*<br>*Aedes mitchellae*<br>*Aedes taeniorhynchus*<br>*Anopheles crucians*<br>*Anopheles punctipennis*<br>*Anopheles quadrimaculatus*<br>*Culex nigripalpus*<br>*Culex salinarius*<br>*Mansonia perturbans*<br>*Psorophora confinnis*

Table S6. Continued

Virus	Virus Family	Virus Genus	Geographic Distribution	Natural Host Family	Arthropod Isolation
West Nile(+)	Flaviviridae	Flavivirus	Mozambique<br>Cent. Af. Rep.<br>Congo<br>Egypt<br>Uganda<br>Nigéria<br>Turkey<br>Cyprus<br>Israel<br>France<br>Portugal<br>Eastern Europe<br>Albania<br>India<br>Malaysia<br>Philippines<br>Thailand	Camelidae<br>Equidae<br>Humans<br>Muridae<br>Other unidentified animals	*Aedes cantans*<br>*Anopheles coustani*<br>*Anopheles subpictus*<br>*Anopheles maculipennis*<br>*Argas hermanni*<br>*Coquillettidia metallica*<br>*Culex antennatus*<br>*Culex modestus*<br>*Culex quinquefasciatus*<br>*Culex univittatus*<br>*Culex vishnui*<br>*Culex weschei*<br>*Hyalomma plumbeum*
Arumowot(-)	Phenuiviridae	Phlebovirus	Cent. Af. Rep.<br>Ethiopia<br>Kenya<br>Nigeria<br>Rhodesia<br>South Africa<br>Sudan	Muridae<br>Turdidae<br>Other unidentified animals	*Culex antennatus*<br>*Culex rubinotus*<br>*Mansonia uniformis*
Bujaru(-)	Phenuiviridae	Phlebovirus	Brazil	Echimyidae	
Bussuquara(-)	Flaviviridae	Flavivirus	Brazil<br>Colombia<br>Panama	Atelidae<br>Echimyidae<br>Humans<br>Other unidentified	*Culex (Mel) sp.*<br>*Culex (Mel) taeniopus*<br>*Culex crybda*<br>*Culex sp.*<br>*Culex vomerifer*<br>*Mansonia titillans*<br>*Trichoprosopon sp.*
Chandipura(-)	Rhabdoviridae	Vesiculovirus	India<br>Nigeria	Bovidae<br>Camelidae<br>Cercopithecid<br>Equidae<br>Erinaceidae<br>Humans	*Phlebotomus sp.*
Chilibre(-)	Phenuiviridae	Phlebovirus	Panama	Unknown	*Lutzomyia ssp.*<br>*Lutzomyia trapidoi*<br>*Lutzomyia ylephilatrix*
Getah(-)	Togaviridae	Alphavirus	Australia<br>Cambodia<br>Japan<br>Malaysia<br>Philippines	Humans	*Aedes vexans nip.*<br>*Anopheles amictus*<br>*Culex (Culex) gelidus*<br>*Culex tritaeniorhynchus*<br>*Culex vishnui*

Table S6. Continued

Virus	Virus Family	Virus Genus	Geographic Distribution	Natural Host Family	Arthropod Isolation
Icoaraci(-)	Phenuiviridae	Phlebovirus	Brazil	Bovidae<br>Cricetidae<br>Echimyidae	Unknown
Ilheus(-)	Flaviviridae	Flavivirus	Argentina<br>Brazil<br>Colombia<br>Guatemala<br>Honduras<br>Panama<br>Trinidad	Bovidae<br>Equidae<br>Suidae<br>Humans<br>Other unidentified	*Aedes ssp.*<br>*Coquillettidia ssp.*<br>*Culex ssp.*<br>*Haemagogus ssp.*<br>*Psorophora ssp.*<br>*Sabethes ssp.*<br>*Trichopropon ssp.*<br>*Wyeomia ssp.*
Itaporanga(-)	Phenuiviridae	Phlebovirus	Brazil<br>French Guiana<br>Trinidad	Didelphidae<br>Humans<br>Muridae<br>Other unidentified	*Coquillettidia venezuelensis*<br>*Culex caudelli*<br>*Culex eastor*
Jamestown canyon(-)	Peribunyaviridae	Orthobunyavirus	USA<br>Canada	Cervidae<br>Humans	*Aedes cantator*<br>*Aedes vexans*<br>*Aedes abserratus*<br>*Aedes communis*<br>*Aedes spp.*<br>*Aedes stimulans*<br>*Aedes triseriatus*<br>*Chrysops cincticornis*<br>*Culiseta inornata*
Japanese Encephalitis(-)	Flaviviridae	Flavivirus	Easter Asia	Ardeidae<br>Equidae<br>Hipposiderida<br>Humans<br>Miniopteridae<br>Suidae	*Anopheles barbirostris*<br>*Anopheles hyrcanus*<br>*Culex annulus*<br>*Culex fuscocephala*<br>*Culex gelidus*<br>*Culex pipiens*<br>*Culex tritaeniorhynchus*<br>*Culex vishnui*
Karimabad(-)	Phenuiviridae	Phlebovirus	Bangladesh<br>Eastern Europe<br>Egypt<br>Iran<br>Pakistan<br>Sudan	Bovidae<br>Columbidae<br>Humans<br>Muridae<br>Phasianidae	*Phlebotomus spp*
Keystone(-)	Peribunyaviridae	Orthobunyavirus	USA	Unknown	*Chrysops obsoletus*
Kokobera(-)	Flaviviridae	Flavivirus	Australia<br>New Guinea	Bovidae<br>Equidae<br>Macropodidae<br>Humans	*Aedes spp.*<br>*Aedes vigilax*<br>*Culex annulirostris*
Kujin(-)	Flaviviridae	Flavivirus	Australia<br>Borneo<br>Indonesia<br>Malaysia<br>New Guinea<br>Sarawak	Bovidae<br>Equidae<br>Humans<br>Oriolidae<br>Phasianidae	*Culex annulirostris*<br>*Culex pseudovishnui*<br>*Culex squamosus*

Table S6. Continued

Virus	Virus Family	Virus Genus	Geographic Distribution	Natural Host Family	Arthropod Isolation
Mayaro(-)	Togaviridae	Alphavirus	USA<br>Bolivia<br>Brazil<br>Colombia<br>Guyana<br>Surinam<br>Trinidad<br>Venezuela	Callitrichidae<br>Cebidae<br>Columbidae<br>Didelphidae<br>Humans<br>Pitheciidae<br>Telidae<br>Tropiduridae<br>Other unidentified	*Culex spp.*<br>*Gigantolaelaps sp.*<br>*Haemagogus spp.*<br>*Mansonia venezuelensis*<br>*Psorophora spp.*<br>*Sabethini spp.*
Oropuche(-)	Peribunyaviridae	Orthobunyavirus	Brazil<br>Colombia<br>Trinidad	Atelidae<br>Bradypodidae<br>Cebidae<br>Humans<br>Other unidentified	*Aedes serratus*<br>*Culex quinquefasciatus*<br>*Culicoides paranensis*<br>*Mansonia venezuelensis*
Orungo(-)	Reoviridae	Orbivirus	Cent. Af. Rep.<br>Central African<br>Ivory Coast<br>Nigeria<br>Senegal<br>Sierra Leone<br>Uganda	Bovidae<br>Camelidae<br>Erinaceidae<br>Humans<br>Soricidae	*Aedes aegypti*<br>*Aedes dentatus*<br>*Anopheles funestus*<br>*Anopheles gambiae*<br>*Culex perfuscus*<br>*Culicoides sp.*
Pacui(-)	Phenuiviridae	Phlebovirus	Brazil<br>Trinidad	Cricetidae<br>Didelphidae<br>Echimyidae<br>Other unidentified	*Lutzomyia flaviscutellata*<br>*Lutzomyia infraspinosa*
Rift Valley Fever(-)	Phenuiviridae	Phlebovirus	Egypt<br>Kenya<br>Nigeria<br>South Africa<br>Sudan<br>Uganda	Bovidae<br>Hipposiderid<br>Humans<br>Pteropodidae<br>Other	*Aedes (Neomelaniconion)*<br>*Aedes (Ochlerotatus) caballus*<br>*Culex (Culex) theileri*<br>*Culicoides spp.*<br>*Eretmapodites chrysogaster*
Ross River(-)	Togaviridae	Alphavirus	American Samoa<br>Australia<br>New Guinea	Canidae<br>Equidae<br>Humans<br>Macropodida<br>Monarchidae<br>Petrocidae<br>Suidae	*Aedes normanensis*<br>*Aedes vigilax*<br>*Aedes vigilax*<br>*Anopheles amictus*<br>*Culex annulirostris*<br>*Culex spp.*<br>*Mansonia uniformis*
Saint Louis encephalitis(-)	Phenuiviridae	Flavivirus	Argentina<br>Brazil<br>Canada<br>Haiti<br>Jamaica<br>Mexico<br>Surinam<br>Trinidad<br>USA<br>Venezuela	Anatidae<br>Apodidae<br>Canidae<br>Cardinalidae<br>Columbidae<br>Turdidae<br>Corvidae<br>Cricetidae<br>Felidae<br>Humans<br>Mimidae<br>Passeridae<br>Pipridae	*Aedes dorsalis*<br>*Anopheles crucians*<br>*Culex caudelli*<br>*Culex coronator*<br>*Culex nigripalpus*<br>*Culex peus*<br>*Culex pipiens*<br>*Culex quinquefasciatus*<br>*Culex spissipes*<br>*Culex taeniopus*<br>*Culex tarsalis*<br>*Culex virgultus*<br>*Deinocerites pseudes*

Table S6. Continued

Virus	Virus Family	Virus Genus	Geographic Distribution	Natural Host Family	Arthropod Isolation
Saint Louis encephalitis(-) (continued)				Thraupidae<br>Other unidentified	*Dermacentor variabilis*<br>*Dermanyssus americanus*<br>*Dermanyssus gallinae*<br>*Gigantolaelaps sp.*<br>*Ornithonysus sylviarum*<br>*Psorophora ferox*<br>*Sabethes belisarioi*<br>*Sabethes chloropterus*<br>*Sabethes ssp.*<br>*Trichopropon sp.*<br>*Wyeomia sp.*
Salehabad(-)	Phenuiviridae	Phlebovirus	Bangladesh<br>Pakistan<br>Iran	Bovidae<br>Columbidae<br>Humans<br>Muridae<br>Passeridae<br>Phasianidae	*Phlebotomus spp.*
San Angelo(-)	Peribunyaviridae	Orthobunyavirus	USA	Canidae<br>Cervidae<br>Didelphidae<br>Humans<br>Phasianidae<br>Procyonidae	*Anopheles pseudopunctipennis*<br>*Psorophora signipennis*
Trivittatus(-)	Peribunyaviridae	Orthobunyavirus	USA	Cricetidae<br>Cervidae	*Aedes atlanticus*<br>*Aedes infirmatus*<br>*Aedes taeniorhynchus*<br>*Aedes triseriatus*<br>*Aedes trivittatus*<br>*Aedes vexas*<br>*Culex pipiens*<br>*Mansonia perturbans*
Uganda S(-)	Flaviviridae	Flavivirus	Cent. Af. Rep.<br>Egypt<br>Gambia<br>India<br>Indonesia<br>Malaysia<br>Mozambique<br>Nigeria<br>Senegal<br>South Africa<br>Tanganyika<br>Uganda	Muscicapidae<br>Humans<br>Other unidentified	*Aedes ingrami*<br>*Aedes longipalpis*<br>*Aedes natronius*
Urucuri(-)	Phenuiviridae	Phlebovirus	Brazil	Dasyproctidae<br>Cricetidae<br>Echimyidae	Unknown
Usutu (+)	Flaviviridae	Flavivirus	South Africa<br>Uganda<br>Nigeria<br>Cent. Af. Rep.<br>Senegal<br>Cameroun	Man<br>Bovidae<br>Muridae<br>Bucerotidae<br>Pycinonotidae<br>Turdidae	*Culex neavei*<br>*Coquillettidia aurites*<br>*Culex pipens*<br>*Culex perfuscus*<br>*Culex perexiguus*<br>*Mansonia africana*<br>*Aedes caspius*

Table S7. List of references used to analyse the vector competence of several mosquito-virus pairs: Aedes aegypti*CHIKV, Aedes aegypti*DENV-1, Aedes aegypti*DENV-2, Aedes aegypti*ZIKV, Aedes albopictus*CHIKV, Aedes albopictus*DENV_1, Aedes albopictus*DENV_2, Aedes albopictus*ZIKV, Culex pipiens*WNV, and Haemagogus leucocelenus*YFV.

**Mosquito-virus pairs**
Ae. aegypti*CHIKV**
Paupy, C.; Ollomo, B.; Kamgang, B.; Moutailler, S.; Rousset, D.; Demanou, M.; Hervé, J.-P.; Leroy, E.; Simard, F. Comparative role of Aedes albopictus and Aedes aegypti in the emergence of Dengue and Chikungunya in central Africa. *Vector Borne Zoonotic Dis.* **2010**, *10*, 259–266.
Vega-Rúa, A.; Lourenço-De-Oliveira, R.; Mousson, L.; Vazeille, M.; Fuchs, S.; Yébakima, A.; Gustave, J.; Girod, R.; Dusfour, I.; Leparc-Goffart, I.; et al. Chikungunya Virus Transmission Potential by Local Aedes Mosquitoes in the Americas and Europe. *PLoS Negl. Trop. Dis.* **2015**, *9*, e0003780.
Vega-Rua, A.; Zouache, K.; Caro, V.; Diancourt, L.; Delaunay, P.; Grandadam, M.; Failloux, A.B. High Efficiency of Temperate Aedes albopictus to Transmit Chikungunya and Dengue Viruses in the Southeast of France. *PLoS One* **2013**, *8*,
Honório, N.A.; Wiggins, K.; Câmara, D.C.P.; Eastmond, B.; Alto, B.W. Chikungunya virus vector competency of Brazilian and Florida mosquito vectors. *PLoS Negl. Trop. Dis.* **2018**, *12*, e0006521.
Ae. aegypti*DENV-1**
Calvez, E.; Guillaumot, L.; Girault, D.; Richard, V.; O'Connor, O.; Paoaafaite, T.; Teurlai, M.; Pocquet, N.; Cao-Lormeau, V.M.; Dupont-Rouzeyrol, M. Dengue-1 virus and vector competence of Aedes aegypti (Diptera: Culicidae) populations from New Caledonia. *Parasites and Vectors* **2017**, *10*, 1–8.
Gonçalves, C.M.; Melo, F.F.; Bezerra, J.M.T.; Chaves, B.A.; Silva, B.M.; Silva, L.D.; Pessanha, J.E.M.; Arias, J.R.; Secundino, N.F.C.; Norris, D.E.; et al. Distinct variation in vector competence among nine field populations of Aedes aegypti from a Brazilian dengue-endemic risk city. *Parasites and Vectors* **2014**, *7*, 320.
Poole-Smith, B.K.; Hemme, R.R.; Delorey, M.; Felix, G.; Gonzalez, A.L.; Amador, M.; Hunsperger, E.A.; Barrera, R. Comparison of Vector Competence of Aedes mediovittatus and Aedes aegypti for Dengue Virus: Implications for Dengue Control in the Caribbean. *PLoS Negl. Trop. Dis.* **2015**, *9*, 1–11.
Richards, S.L.; Anderson, S.L.; Alto, B.W. Vector Competence of Aedes aegypti and Aedes albopictus (Diptera: Culicidae) for Dengue Virus in the Florida Keys . *J. Med. Entomol.* **2012**, *49*, 942–946.
Ae. aegypti*DENV-2**
Chepkorir, E.; Lutomiah, J.; Mutisya, J.; Mulwa, F.; Limbaso, K.; Orindi, B.; Ng'Ang'a, Z.; Sang, R. Vector competence of Aedes aegypti populations from Kilifi and Nairobi for dengue 2 virus and the influence of temperature. *Parasites and Vectors* **2014**, *7*, 435.
Diallo, M.; Ba, Y.; Faye, O.; Soumare, L.; Dia, I.; Sall, A.A. Vector competence of Aedes aegypti populations from Senegal for sylvatic and epidemic dengue 2 virus isolated in West Africa. *Trans. R. Soc. Trop. Med. Hyg.* **2008**, *102*, 493–498.
Gonçalves, C.M.; Melo, F.F.; Bezerra, J.M.T.; Chaves, B.A.; Silva, B.M.; Silva, L.D.; Pessanha, J.E.M.; Arias, J.R.; Secundino, N.F.C.; Norris, D.E.; et al. Distinct variation in vector competence among nine field populations of Aedes aegypti from a Brazilian dengue-endemic risk city. *Parasites and Vectors* **2014**, *7*, 320.
Poole-Smith, B.K.; Hemme, R.R.; Delorey, M.; Felix, G.; Gonzalez, A.L.; Amador, M.; Hunsperger, E.A.; Barrera, R. Comparison of Vector Competence of Aedes mediovittatus and Aedes aegypti for Dengue Virus: Implications for Dengue Control in the Caribbean. *PLoS Negl. Trop. Dis.* **2015**, *9*, e0003462.
Ae. aegypti*ZIKV**
Amraoui, F.; Ayed, W. Ben; Madec, Y.; Faraj, C.; Himmi, O.; Btissam, A.; Sarih, M.; Failloux, A.B. Potential of aedes albopictus to cause the emergence of arboviruses in Morocco. PLoS Negl. Trop. Dis. 2019, 13, e0006997.
Bonica, M.B.; Goenaga, S.; Martin, M.L.; Feroci, M.; Luppo, V.; Muttis, E.; Fabbri, C.; Morales, M.A.; Enria, D.; Micieli, M.V.; et al. Vector competence of Aedes aegypti for different strains of Zika virus in Argentina. *PLoS Negl. Trop. Dis.* **2019**, *13*, e0007433.
Calvez, E.; O'Connor, O.; Pol, M.; Rousset, D.; Faye, O.; Richard, V.; Tarantola, A.; Dupont-Rouzeyrol, M. Differential transmission of Asian and African Zika virus lineages by Aedes aegypti from New Caledonia. *Emerg. Microbes Infect.* **2018**, *7*, 159.
Chouin-Carneiro, T.; Vega-Rua, A.; Vazeille, M.; Yebakima, A.; Girod, R.; Goindin, D.; Dupont-Rouzeyrol, M.; Lourenço-de-Oliveira, R.; Failloux, A.B. Differential Susceptibilities of Aedes aegypti and Aedes albopictus from the Americas to Zika Virus. *PLoS Negl. Trop. Dis.* **2016**, *10*, e4543.
Hall-Mendelin, S.; Pyke, A.T.; Moore, P.R.; Ritchie, S.A.; Moore, F.A.J.; Van Den Hurk, A.F. Characterization of a Western Pacific Zika Virus Strain in Australian Aedes aegypti. *Vector-Borne Zoonotic Dis.* **2018**, *18*, 317–322.

Table S7. Continued

**Mosquito-virus pairs**
Ae. aegypti***ZIKV** (continued)
Heitmann, A.; Jansen, S.; Lühken, R.; Leggewie, M.; Badusche, M.; Pluskota, B.; Becker, N.; Vapalahti, O.; Schmidt-Chanasit, J.; Tannich, E. Experimental transmission of zika virus by mosquitoes from central Europe. *Eurosurveillance* **2017**, *22*, 14–17.
Hery, L.; Boullis, A.; Delannay, C.; Vega-Rúa, A. Transmission potential of African, Asian and American Zika virus strains by Aedes aegypti and Culex quinquefasciatus from Guadeloupe (French West Indies). *Emerg. Microbes Infect.* **2019**, *8*, 699–706.
Hugo, L.E.; Stassen, L.; La, J.; Gosden, E.; Ekwudu, O.; Winterford, C.; Viennet, E.; Faddy, H.M.; Devine, G.J.; Frentiu, F.D. Vector competence of Australian Aedes aegypti and Aedes albopictus for an epidemic strain of Zika virus. *PLoS Negl. Trop. Dis.* **2019**, *13*, e0007281.
Liu, Z.; Zhou, T.; Lai, Z.; Zhang, Z.; Jia, Z.; Zhou, G.; Williams, T. Competence of Aedes aegypti, Aedes albopictus, and Culex quinquefasciatus mosquitoes as Zika Virus Vectors, China. *Emerg. Infect. Dis.* **2017**, *23*, 1085–1091.
Luca, M. Di; Severini, F.; Toma, L.; Boccolini, D.; Romi, R.; Remoli, M.E.; Sabbatucci, M.; Rizzo, C.; Venturi, G.; Rezza, G. Experimental studies of susceptibility of Italian Aedes albopictus to Zika virus. Euro Surveill. 2016, 21, 18.
Main, B.J.; Nicholson, J.; Winokur, O.C.; Steiner, C.; Riemersma, K.K.; Stuart, J.; Takeshita, R.; Krasnec, M.; Barker, C.M.; Coffey, L.L. Vector competence of Aedes aegypti, Culex tarsalis, and Culex quinquefasciatus from California for Zika virus. *PLoS Negl. Trop. Dis.* **2018**, *12*, e0006524.
Richard, V.; Paoaafaite, T.; Cao-Lormeau, V.M. Vector Competence of French Polynesian Aedes aegypti and Aedes polynesiensis for Zika Virus. *PLoS Negl. Trop. Dis.* **2016**, *10*, e0005024.
Aedes aegypti***YFV**
Couto-Lima, Di.; Madec, Y.; Bersot, M.I.; Campos, S.S.; Motta, M.D.A.; Dos Santos, F.B.; Vazeille, M.; Da Costa Vasconcelos, P.F.; Lourenço-De-Oliveira, R.; Failloux, A.B. Potential risk of re-emergence of urban transmission of Yellow Fever virus in Brazil facilitated by competent Aedes populations. *Sci. Rep.* **2017**, *7*, 4848.
Johnson, B.; Chambers, T.; Grabtree, M.; Filippis, A.; Vilarinhos, P.; Resende, M.; Marcoris, L.; Miller, B. Vector competence of Brazilian Aedes aegypti and Ae . abopictus for a Brazilian yellow fever virus isolate *. *Trans. R. Soc. Trop. Med. Hyg.* **2002**, *96*, 611–613.
Lourenço de Oliveira, R.; Vazeille, M.; Maria, A.N.A.; Filippis, B.D.E.; Failloux, A. Large genetic differentiation and low variation in vector competence for dengue and yellow fever viruses of Aedes albopictus from Brazil, the United States, and the Cayman Islands. *Am. J. Trop. Med. Hyg.* **2003**, *69*, 105–114.
Ellis, B.R.; Sang, R.C.; Horne, K.M.; Higgs, S.; Wesson, D.M. Yellow fever virus susceptibility of two mosquito vectors from Kenya, East Africa. *Trans. R. Soc. Trop. Med. Hyg.* **2012**, *106*, 387–389.
Yen, P.S.; Amraoui, F.; Rúa, A.V.; Failloux, A.B. Aedes aegypti mosquitoes from guadeloupe (French west indies) are able to transmit yellow fever virus. *PLoS One* **2018**, *13*, e0204710
Van Den Hurk, A.F.; McElroy, K.; Pyke, A.T.; McGee, C.E.; Hall-Mendelin, S.; Day, A.; Ryan, P.A.; Ritchie, S.A.; Vanlandingham, D.L.; Higgs, S. Vector competence of Australian mosquitoes for yellow fever virus. *Am. J. Trop. Med. Hyg.* **2011**, *85*, 446–451.
Jupp, P.G.; Kemp, A. Laboratory vector competence experiments with yellow fever virus and five South African mosquito species including Aedes aegypti. *Trans R Soc Trop Med Hyg* **2002**, *96*, 493–498.
Aedes albopictus***CHIKV**
Amraoui, F.; Ayed, W. Ben; Madec, Y.; Faraj, C.; Himmi, O.; Btissam, A.; Sarih, M.; Failloux, A.B. Potential of Aedes albopictus to cause the emergence of arboviruses in Morocco. *PLoS Negl. Trop. Dis.* **2019**, *13*, e0006997.
Fortuna, C.; Toma, L.; Remoli, M.E.; Amendola, A.; Severini, F.; Boccolini, D.; Romi, R.; Venturi, G.; Rezza, G.; Di Luca, M. Vector competence of Aedes albopictus for the Indian Ocean Lineage (IOL) chikungunya viruses of the 2007 and 2017 outbreaks in italy: A comparison between strains with and without the E1:A226V mutation. *Eurosurveillance* **2018**, *23*, 1800246.
Honório, N.A.; Wiggins, K.; Câmara, D.C.P.; Eastmond, B.; Alto, B.W. Chikungunya virus vector competency of Brazilian and Florida mosquito vectors. *PLoS Negl. Trop. Dis.* **2018**, *12*, e0006521.
Ngoagouni, C.; Kamgang, B.; Kazanji, M.; Paupy, C.; Nakouné, E. Potential of Aedes aegypti and Aedes albopictus populations in the Central African Republic to transmit enzootic chikungunya virus strains. *Parasites and Vectors* **2017**, *10*, 164.
Paupy, C.; Ollomo, B.; Kamgang, B.; Moutailler, S.; Rousset, D.; Demanou, M.; Hervé, J.-P.; Leroy, E.; Simard, F. Comparative role of Aedes albopictus and Aedes aegypti in the emergence of Dengue and Chikungunya in central Africa. *Vector Borne Zoonotic Dis.* **2010**, *10*, 259–266.
Vega-Rua, A.; Zouache, K.; Caro, V.; Diancourt, L.; Delaunay, P.; Grandadam, M.; Failloux, A.B. High Efficiency of Temperate Aedes albopictus to Transmit Chikungunya and Dengue Viruses in the Southeast of France. *PLoS One* **2013**, *8*, e59716.
Vega-Rúa, A.; Lourenço-De-Oliveira, R.; Mousson, L.; Vazeille, M.; Fuchs, S.; Yébakima, A.; Gustave, J.; Girod, R.; Dusfour, I.; Leparc-Goffart, I.; et al. Chikungunya Virus Transmission Potential by Local Aedes Mosquitoes in the Americas and

Table S7. Continued

**Mosquito-virus pairs**
***Aedes albopictus**DENV-1**
Brustolin, M.; Santamaria, C.; Napp, S.; VerdÚn, M.; Rivas, R.; Pujol, N.; Talavera, S.; Busquets, N. Experimental study of the susceptibility of a European Aedes albopictus strain to dengue virus under a simulted Mediterranean temperature regime. *Med. Vet. Entomol.* **2018**, *32*, 393–398.
Richards, S.L.; Anderson, S.L.; Alto, B.W. Vector Competence of Aedes aegypti and Aedes albopictus (Diptera: Culicidae) for Dengue Virus in the Florida Keys . *J. Med. Entomol.* **2012**, *49*, 942–946.
***Aedes albopictus**DENV-2**
Amraoui, F.; Ayed, W. Ben; Madec, Y.; Faraj, C.; Himmi, O.; Btissam, A.; Sarih, M.; Failloux, A.B. Potential of aedes albopictus to cause the emergence of arboviruses in Morocco. PLoS Negl. Trop. Dis. **2019**, 13, e0006997.
Brustolin, M. et al. Experimental study of the susceptibility of a European Aedes albopictus strain to dengue virus under a simulted Mediterranean temperature regime. *Med. Vet. Entomol*. **2018**, 32, 393–398.
Paupy, C. et al. Comparative role of Aedes albopictus and Aedes aegypti in the emergence of Dengue and Chikungunya in central Africa. *Vector Borne Zoonotic Dis*. **2010**, 10, 259–266.
***Aedes albopictus**ZIKV**
Amraoui, F.; Ayed, W. Ben; Madec, Y.; Faraj, C.; Himmi, O.; Btissam, A.; Sarih, M.; Failloux, A.B. Potential of aedes albopictus to cause the emergence of arboviruses in Morocco. PLoS Negl. Trop. Dis. **2019**, 13, e0006997.
Heitmann, A.; Jansen, S.; Lühken, R.; Leggewie, M.; Badusche, M.; Pluskota, B.; Becker, N.; Vapalahti, O.; Schmidt-Chanasit, J.; Tannich, E. Experimental transmission of zika virus by mosquitoes from central Europe. Eurosurveillance **2017**, 22, 14–17.
Hugo, L.E.; Stassen, L.; La, J.; Gosden, E.; Ekwudu, O.; Winterford, C.; Viennet, E.; Faddy, H.M.; Devine, G.J.; Frentiu, F.D. Vector competence of Australian Aedes aegypti and Aedes albopictus for an epidemic strain of Zika virus. PLoS Negl. Trop. Dis. **2019**, 13, e0007281.
Liu, Z.; Zhou, T.; Lai, Z.; Zhang, Z.; Jia, Z.; Zhou, G.; Williams, T. Competence of Aedes aegypti, Aedes albopictus, and Culex quinquefasciatus mosquitoes as Zika Virus Vectors, China. Emerg. Infect. Dis. **2017**, 23, 1085–1091.
Luca, M. Di; Severini, F.; Toma, L.; Boccolini, D.; Romi, R.; Remoli, M.E.; Sabbatucci, M.; Rizzo, C.; Venturi, G.; Rezza, G. Experimental studies of susceptibility of Italian Aedes albopictus to Zika virus. Euro Surveill. **2016**, 21, 18.
González, M.A.; Pavan, M.G.; Fernandes, R.S.; Busquets, N.; David, M.R.; Lourenço-Oliveira, R.; García-Pérez, A.L.; MacIel-De-Freitas, R. Limited risk of Zika virus transmission by five Aedes albopictus populations from Spain. Parasites and Vectors **2019**, 12, 150.
***Culex pipiens**WNV**
Brustolin, M.; Talavera, S.; Nunez, A.; SantamarÍa, C.; Rivas, R.; Pujol, N.; Valle, M.; Verdun, M.; Brun, A.; Pages, N.; et al. Rift Valley fever virus and European mosquitoes: vector competence of Culex pipiens and Stegomyia albopicta (= Aedes albopictus). *Med. Vet. Entomol.* **2017**, 31, 365–372.
Fortuna, C.; Remoli, M.E.; Di Luca, M.; Severini, F.; Toma, L.; Benedetti, E.; Bucci, P.; Montarsi, F.; Minelli, G.; Boccolini, D.; et al. Experimental studies on comparison of the vector competence of four Italian Culex pipiens populations for West Nile virus. *Parasit. Vectors* **2015**, *8*, 463.
Fros, J.J.; Miesen, P.; Vogels, C.B.; Gaibani, P.; Sambri, V.; Martina, B.E.; Koenraadt, C.J.; van Rij, R.P.; Vlak, J.M.; Takken, W.; et al. Comparative Usutu and West Nile virus transmission potential by local Culex pipiens mosquitoes in north-western Europe. *One Heal.* **2015**, *1*, 31–36.
Goddard, L.B.; Roth, A.E.; Reisen, W.K.; Scott, T.W.; States, U. Vector Competence of California Mosquitoes for. *Emerg. Infect. Dis.* **2002**, *8*, 1385–1391.
Ebel, G.D.; Rochlin, I.; Longacker, J.; Kramer, L.D. Culex restuans (Diptera: Culicidae) relative abundance and vector competence for West Nile Virus. *J. Med. Entomol.* **2005**, 42, 838–843.
Kilpatrick, A.; Fonseca, D.M.; Ebel, G.D.; Reddy, M.R.; Kramer, L.D. Spatial and temporal variation in vector competence of Culex pipiens and Cx. restuans mosquitoes for West Nile virus. *Am. J. Trop. Med. Hyg.* **2010**, 83, 607–613.
Richards, S.L.; Mores, C.N.; Lord, C.C.; Tabachnick, W.J. Impact of Extrinsic Incubation Temperature and Virus Exposure on Vector Competence of *Culex pipiens quinquefasciatus* Say (Diptera: Culicidae) for West Nile Virus. *Vector-Borne Zoonotic Dis.* **2007**, 7, 629–636.
Richards, S.L.; Anderson, S.L.; Lord, C.C. Vector competence of Culex pipiens quinquefasciatus (Diptera: Culicidae) for West Nile virus isolates from Florida. *Trop. Med. Int. Heal.* **2014**, 19, 610–617.
Anderson, S.L.; Richards, S.L.; Tabachnick, W.J.; Smartt, C.T. Effects of West Nile Virus Dose and Extrinsic Incubation Temperature on Temporal Progression of Vector Competence in Culex pipiens quinquefasciatus. *J. Am. Mosq. Control Assoc.* **2010**, 26, 103–107.
Turell, M.J.; Guinn, M.L.O.; Dohm, D.J.; Jones, J.W. Vector Competence of North American Mosquitoes (Diptera®: Culicidae) for West Nile Virus. *J. Med. Entomol.* **2001**, 38, 130–134.

Table S7. Continued

**Mosquito-virus pairs**
***Culex pipiens**WNV** (continued)
Vanlandingham, D.L.; McGee, C.E.; Klinger, K.A.; Vessey, N.; Fredregillo, C.; Higgs, S. Short report: Relative susceptibilties of South Texas mosquitoes to infection with West Nile virus. Am. J. Trop. Med. Hyg. 2007, 77, 925–928.
Vaidyanathan, R.; Fleisher, A.E.; Minnick, S.L.; Simmons, K.A.; Scott, T.W. Nutritional Stress Affects Mosquito Survival and Vector Competence for West Nile Virus. Vector-Borne Zoonotic Dis. 2008, 8, 727–732.
***Haemagogus leucocelenus**YFV**
Couto-Lima, Di.; Madec, Y.; Bersot, M.I.; Campos, S.S.; Motta, M.D.A.; Dos Santos, F.B.; Vazeille, M.; Da Costa Vasconcelos, P.F.; Lourenço-De-Oliveira, R.; Failloux, A.B. Potential risk of re-emergence of urban transmission of Yellow Fever virus in Brazil facilitated by competent Aedes populations. *Sci. Rep.* **2017**, *7*, 4848.

Figure S1. Analysis of the host feeding patterns of *Ae. albopictus* for the different species of domestic animals without taking into account the host availability. Black line: median.

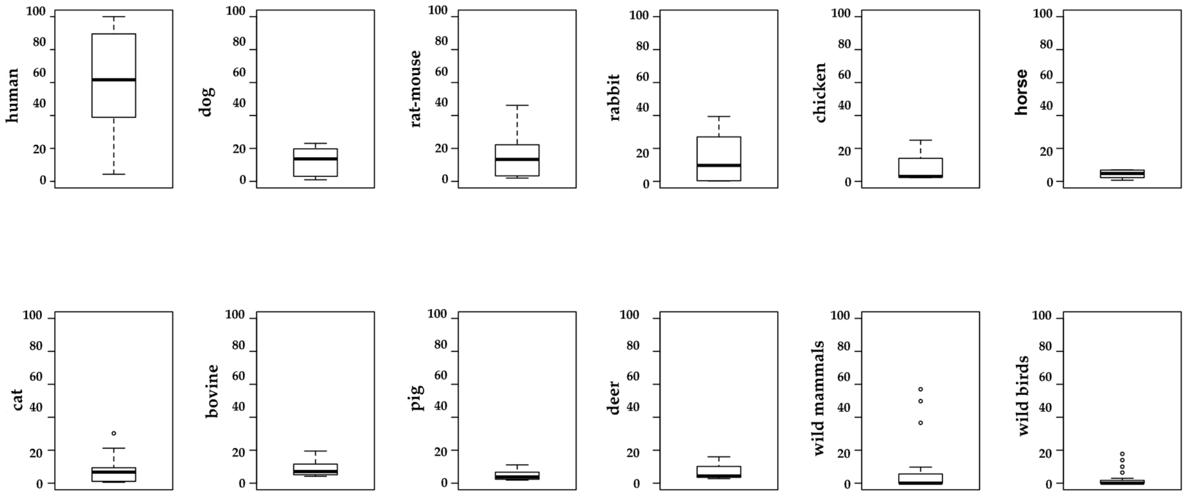

Supplementary Material and Methods.

Estimating the relative virus transfer risk mediated by *Ae. albopictus*.

We calculated the relative transfer risk of viruses mediated by *Ae. albopictus* from their natural host to humans by adapting Kilpatrick's equation: *Risk = A x P x Fm x Cv*, where *A* corresponds to the mosquito abundance, *P* is the virus infection prevalence, *Fm* is the fraction of blood meals taken from mammals, and *Cv* corresponds to the fraction of infected mosquitoes that can transmit the virus in a subsequent bite. In our study and for all viruses, information on *A* and *P* were lacking, but as these two parameters are directly proportional fractions of the formula (i.e. the more we increase their value, the greater the risk is) we assumed that the absence of information should not affect the result of the risk assessment. We decomposed *Fm* in *FAi* (i.e. the mean relative frequency *Ae. albopictus* bites in vertebrate host families) and *FHi* (i.e. the mean relative frequency of *Ae. albopictus* bites in humans). We then calculated the average *FAi* and *FHi* using the information on *Ae. albopictus* feeding behavior extracted from the articles identified in our literature search. For such purpose, we listed all the natural vertebrate hosts of each virus using the information available in the CDC catalogue and then classified hosts according to their family. We calculated the mean frequency of *Ae. albopictus* biting for each family, when data were available. *Cv* is equal to the Transmission efficiency (TRi). In conclusion, for each virus in the present study, the risk assessment was calculated as TRi x FAi x FHi. The results are presented in Figure S2, but are not included in the main text due to the uncertainties in the calculation because of the limitations of the representative data on feeding behaviour, arbovirus hosts and vector competence to produce quantitative estimations and extrapolations

Figure S2. Relative virus transfer risk by *Ae. albopictus* for 13 enzootic viruses experimentally proven to be transmitted by *Ae. albopictus* and for four epidemic arboviruses (DENV-1, DENV-2, ZIKAV, and CHIKV).

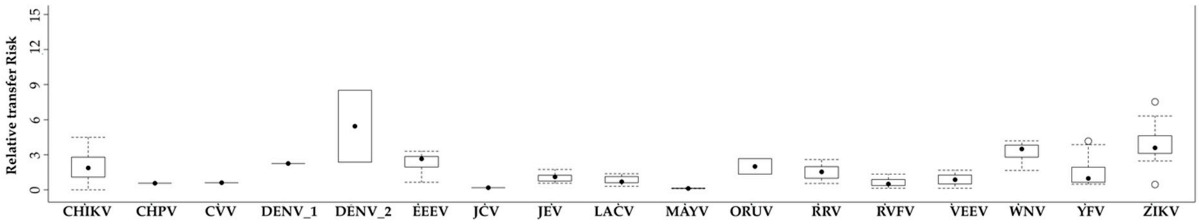

CHIKV, Chikungunya virus; CHPV, Chandipura virus; CVV, Cache Valley virus; DENV-1, Dengue virus serotype 1; DENV-2, Dengue virus serotype 2; EEEV, Eastern equine encephalomyelitis virus; JCV, Jamestown Canyon virus; JEV, Japanese encephalitis virus; LACV, La Crosse virus; MAYV, Mayaro virus; ORUV, Orungo virus; RRV, Ross River virus; RVFV, Rift Valley fever virus; VEEV, Venezuelan equine encephalitis virus; WNV, West Nile virus; YFV, Yellow fever virus; and ZIKV, Zika virus.

© 2020 by the authors. Submitted for possible open access publication under the terms and conditions of the Creative Commons Attribution (CC BY) license (http://creativecommons.org/licenses/by/4.0/).
